# Human mobility increased with vaccine coverage and attenuated the protection of COVID-19 vaccination: A longitudinal study of 107 countries

**DOI:** 10.7189/jogh.13.06009

**Published:** 2023-04-07

**Authors:** Li-Lin Liang, Huong Mai Le, Chun-Ying Wu, Chien-Yuan Sher, Alistair McGuire

**Affiliations:** 1 Institute of Public Health, National Yang Ming Chiao Tung University, Taipei, Taiwan; 2 Research Center for Epidemic Prevention and One Health, National Yang Ming Chiao Tung University, Taipei, Taiwan; 3 Health Innovation Centre, National Yang Ming Chiao Tung University, Taipei, Taiwan; 4 Department of Business Management, National Sun Yat-sen University, Kaohsiung, Taiwan; 5 Faculty of Economics, National Economics University, Hanoi, Vietnam; 6 Institute of Biomedical Informatics, National Yang Ming Chiao Tung University, Taipei, Taiwan; 7 Microbiota Research Centre, National Yang Ming Chiao Tung University, Taipei, Taiwan; 8 Division of Translational Research, Taipei Veterans General Hospital, Taipei, Taiwan; 9 Department of Public Health, China Medical University, Taichung, Taiwan; 10 Department of Health Policy, London School of Economics and Political Science, London, UK

## Abstract

**Background:**

The World Health Organization has raised concerns that vaccinated people may reduce physical and social distancing more than necessary. With imperfect vaccine protection and the lifting of mobility restrictions, understanding how human mobility responded to vaccination and its potential consequence is critical. We estimated vaccination-induced mobility (VM) and examined whether it attenuates the effect of COVID-19 vaccination on controlling case growth.

**Methods:**

We collected a longitudinal data set of 107 countries between 15 February 2020 and 6 February 2022 from Google COVID-19 Community Mobility Reports, the Oxford COVID-19 Government Response Tracker, Our World in Data, and World Development Indicators. We measured mobility in four categories of location: retail and recreational places, transit stations, grocery stores and pharmacies, and workplaces. We applied panel data models to address unobserved country characteristics and used Gelbach decomposition to evaluate the extent to which VM has offset vaccination effectiveness.

**Results:**

Across locations, a 10-percentage-point (pp) increase in vaccine coverage was associated with a 1.4-4.3 pp increase in mobility (*P* < 0.001). VM was greater in lower-income countries (up to 7.9 pps; 95% confidence interval (CI) = 5.3 to 10.5, *P* < 0.001) and in earlier stages of vaccine rollouts (up to 19.2 pps; 95% CI = 15.1 to 23.2%, *P* < 0.001). VM decreased the effectiveness of vaccines in controlling case growth by 33.4% in retail and recreation places (*P* < 0.001), 26.4% in transit stations (*P* < 0.001), and 15.4% in grocery stores and pharmacies (*P* = 0.002).

**Conclusions:**

VM provides support for the Peltzman effect; it attenuates but does not completely counter vaccine effectiveness. Our study findings suggest strategies for mitigating the unintended consequences of VM, including reducing short-term mobility responses after vaccination, prioritizing mobility in grocery-type places and workplaces, and accelerating rollouts at earlier stages of vaccination, especially in lower-income countries.

Clinical trials have demonstrated the efficacy of vaccines against the severe acute respiratory syndrome coronavirus 2 (SARS-CoV-2) [1]. However, whether coronavirus disease 2019 (COVID-19) vaccination has achieved its full benefits in the real world remains unclear. One reason for this is that people may adjust their behaviours after vaccination. Concerns have been raised regarding the possibility that vaccinated individuals reduce adherence to preventive measures, which can be explained by the Peltzman effect [2-4]. This effect, originally proposed in the context of automobile safety regulation, hypothesises that people take greater risks when they feel protected and behave more carefully when they perceive a higher level of risk [5]. In the context of COVID-19 vaccination, lowered perceived risk of infection among the public may lead to reductions in social and physical distancing. This is concerning because vaccine effectiveness has waned over time [6], and breakthrough infections have continued to rise [7]. The World Health Organization has strongly urged people to continue maintaining social distancing and avoiding crowds [8]. To address this, understanding how human mobility responds to vaccination is critical for post-vaccination pandemic control.

Research on mobility adjustments after COVID-19 vaccination is scarce. To our knowledge, only six studies using survey data have been conducted on this topic. Research conducted in 12 high-income countries found that individuals who received two vaccine doses less frequently practised physical distancing than those who received one or no doses [9]. Moreover, a Bangladesh study conducted from July to August 2021 reported that vaccinated individuals increased physical contact with unvaccinated individuals, visited crowded places, travelled, and stayed outside longer than unvaccinated individuals [10]. A UK survey from June 2021 showed that vaccinated individuals participated in more social activities, such as visiting pubs, restaurants, theatres, and cinemas, and that they exhibited reduced precautionary avoidance of crowded spaces [11]. Additionally, a UK survey conducted in February 2021 reported that 41% of respondents who had received one dose of a vaccine increased their social contacts, which contradicted lockdown-regulations [12]. However, another UK survey and a US study did not reveal changes in social distancing after vaccination prior to March 2021 [13,14]. Based on survey evidence, the UK Scientific Advisory Group demonstrated the importance of better understanding behavioural changes potentially caused by the vaccine rollout [15].

To fill this research gap, we investigated the complex relationship between mobility, COVID-19 vaccination, and case growth. We aimed to estimate mobility changes attributable to the expansion of vaccine coverage, termed vaccination-induced mobility (VM), and to quantify the extent to which they offset the effect of COVID-19 vaccination on controlling case growth.

This study is original in that it provides empirical evidence on behavioural adjustments in response to COVID-19 vaccination and quantifies the unintended consequences of VM on vaccine effectiveness. SARS-CoV-2 will continue to cause waves of infection while constantly mutating [16]. With the lifting of lockdowns and limited protection offered by vaccines, behavioural responses will profoundly affect the course of the pandemic.

## METHODS

**Figure 1:**
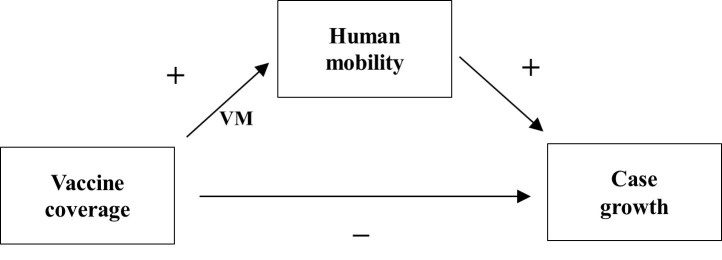
Associations among human mobility, vaccine coverage, and COVID-19 case growth. VM represents vaccination-induced mobility. A +/– sign denotes a positive/negative association.

Although vaccine effectiveness against COVID-19 is well documented [17,18], mobility has often been excluded from studies that analysed vaccine-infection association. We hypothesise that a vaccinated individual may increase their mobility after vaccination, thus becoming more susceptible to infection than an unvaccinated individual who refrains from going around. Consequently, the VM may have weakened the expected benefits of mass vaccination, so we proposed a conceptual framework, illustrated in **Figure 1**.

In these hypothesised relationships, vaccine coverage is expected to reduce case growth, and mobility is expected to increase case growth. Accordingly, we hypothesised that mobility mediates the relationship between COVID-19 vaccination and infection, weakening the negative association of vaccine coverage with case growth.

### Data sources and study sample

We collected data from the following open-access databases for the period from 15 February 2020 to 6 February 2022: Google Community Mobility Report [19], the Oxford COVID-19 Government Response Tracker (OxCGRT) [20], Our World in Data (OWID) [21], and World Development Indicators [22], as summarized in **Table 1**. The exclusion criteria were as follows: having a vaccination programme in place for less than one year by February 2022 and being classified as a low-income country by the World Bank based on 2020 per capita gross national income (US$1045). After applying exclusion criteria, the final sample was a longitudinal data set of 107 countries with approximately 75 600 country-days. A flowchart of the sample selection process and a list of sample countries are presented in Appendix S1 and S2 of the **Online Supplementary Document**.

**Table 1:** Summary of data sources

Database	Variables
Google Community Mobility Report	Human mobility
The Oxford COVID-19 Government Response Tracker	Nonpharmaceutical interventions
Our World in Data	Growth of COVID-19 cases, COVID-19 vaccine coverage, and risk information (as measured by the lagged number of new COVID-19 cases)
World Development Indicators	Country characteristics, i.e., population density, proportion of population aged ≥65 y, per capita gross domestic product, the Gini index, female proportion

### Empirical models and estimation

We conducted a two-stage empirical analysis to examine the mediating role of mobility. In the first stage, we developed a mobility model to estimate VM; this model used vaccine coverage as the key predictor and mobility as the outcome. In the second stage, we treated the case growth as the outcome variable and developed the base and full models. The base model used only vaccine coverage as the key predictor, while the full model used both vaccine coverage and human mobility. We evaluated the extent to which mobility mediated the vaccine-infection relationship as the change in vaccine coverage coefficient between the base and full models. If mobility mediated the relationship between vaccination and case growth, then the negative vaccine coverage coefficient would become smaller or exhibit a downward bias (in absolute value) when mobility was excluded from the model.

We applied panel data models in both stages to account for the time-invariant unobservable country effects, such as differences in national culture and COVID-19 surveillance systems [23]. We assumed random effects for the mobility model and fixed effects for the case growth model. Empirical models and estimation strategies are discussed in Appendix S3 in the **Online Supplementary Document**. Notably, we used only post-vaccination data in the case growth model, defining the postvaccination period as the days since the vaccine rollout began. Because the countries introduced COVID-19 vaccination programmes at different times, we used government websites and international statistics to identify the first day of vaccine rollout. The post-vaccination period comprised approximately 52% of the country-days in our full sample. Additionally, we estimated the difference in vaccine coverage coefficient between the base and full models using the decomposition method proposed by Gelbach [24]. The derivation of the decomposition formula is described in Appendix S4 in the **Online Supplementary Document**. We performed all analyses using Stata 17 (StataCorp, College Station, TX, USA).

### Variables

#### Human mobility

We utilised the daily data in the COVID-19 Community Mobility Report to assess human mobility. In this database, mobility is recorded for six categories of location: retail and recreation, grocery stores and pharmacies, transit stations, workplaces, parks, and places of residence. We analysed the first four categories in this study. The baseline value for mobility on a particular day was the median value for the corresponding day of the week during the five-week period from 3 January to 6 February 2020. We evaluated mobility as the percentage change in visits for a particular day relative to the baseline or normal value for that day of the week. When only subnational data were available, we calculated mobility by averaging the mobility data for each day among all regions in a country. To incorporate all four mobility variables, we calculated their arithmetic mean and created a variable called mobility index.

#### Growth of COVID-19 cases

Following the approach described by Hsiang et al. [25], we focused on the growth of active infections. Using the susceptible–infectious–removed disease model, Hsiang et al. derived a case growth model and assumed that case growth is a linear function of time-varying covariates. We calculated case growth as follows:

*casegrowth_i,t_* = ((*y_i.t._ − y_i.t. −1_*)/*y_i.t. −1_*) × 100

where y_i.t._ is a seven-day moving average of new cases on day *t* in country *i*. We used a seven-day moving average to reduce systematic fluctuations in reported case numbers. Data on total cases were obtained from OWID, a database established by the Global Change Data Laboratory in partnership with the University of Oxford [21].

#### COVID-19 vaccine coverage

We extracted data for COVID-19 vaccine coverage (or vaccination rate) from the OWID database. We defined coverage as the cumulative number of people who received at least one dose of a COVID-19 vaccine per 10 people per day. We filled any gaps in the data by using a linear interpolation model and assuming that the vaccination rate is a function of time. For the mobility model, we used the present value of vaccine coverage. For the case growth model, we used the effective coverage rate, calculated as the cumulative number of people who received at least one dose of COVID-19 vaccines per 10 people from seven days and six months ago. Protection against COVID-19 infection has been reported to wane after six months [26]. Accordingly, we focused on the number of people vaccinated in the recent six months.

#### Control variables

We measured non-pharmaceutical interventions (NPIs) using the stringency index published by OxCGRT, which is a database that tracks government responses to the COVID-19 outbreak across countries over time [20]. The stringency index is a composite measure of nine NPIs: school closures, workplace closures, cancellation of public events, restrictions on gatherings, public transport closures, stay-at-home requirements, restrictions on internal movement, restrictions on international travel, and public information campaigns. The index is a continuous value that ranges from 0 to 100, with a higher value indicating greater intensity of policy measures imposed by a country. We used this index in both mobility and case growth models.

For case growth model, we considered another NPI in the OxCGRT database, which is the requirement of face coverings. We rated this variable on an ordinal scale with the values of 0, 1, 2, 3, and 4, with a value of 0 denoting “no requirement” and a value of 4 denoting that face coverings were “required in all places at all times”. We transformed this variable into five dummy variables.

We measured risk information using the number of new confirmed cases of COVID-19 per 10 000 people per country per day, only including it in the mobility model to capture changes in risk perceptions caused by factors other than COVID-19 vaccination. Relevant studies have demonstrated that information on COVID-19 infections contributed to decreases in mobility [27,28]. Accordingly, we lagged this variable by one day to account for time for behavioural response. Additionally, we included a comprehensive set of country characteristics and time factors as control variables, as discussed in Appendix S2 in the **Online Supplementary Document**.

### Subgroup analysis and robustness checks

Income level and socioeconomic status have been reported to influence mobility [29,30]. At the aggregate level, countries at different levels of development may exhibit different degrees of human mobility. To investigate whether the association between mobility and vaccine coverage varied with development stage, we classified 107 countries into high-income (HI) (50 countries), upper-middle-income (UMI) (30 countries), and lower-middle-income (LMI) (27 countries) categories on the basis of 2020 per capita gross national income threshold specified by the World Bank [31]. Subsequently, we performed mobility regression analyses for individual income groups.

We conducted a series of robustness checks for mobility models (Appendix S5 in the **Online Supplementary Document**). The estimation strategies were: replacing country random effects with fixed effects, replacing mobility outcome with its seven-day moving average, lagging vaccine coverage by seven days, using instrumental variables for vaccine coverage, and adding the number of boosters and interaction with vaccine coverage (Tables S5.1 to S5.5 in the **Online Supplementary Document**). For the case growth model, we replaced the growth outcome with the reproduction rate and presented the results in Table S6.1, Appendix S6 in the **Online Supplementary Document**. Additionally, we included the number of different COVID-19 vaccines (Pfizer/BioNTech, Moderna, and Oxford/AstraZeneca) (Table S6.4 in the **Online Supplementary Document****)**.

## RESULTS

### Summary of model variables

**Table 2** presents a summary of the model variables. For the mobility model, compared with pre-pandemic values, the mean number of visitors decreased by 13.2% for retail and recreation (termed retail stores hereafter), 18.9% for transit stations, and 17.6% for workplaces. By contrast, the total number of visitors increased by 8.3% for grocery stores and pharmacies (termed grocery stores hereafter). A mean 1.8 people per 10 (18% coverage rate) received at least one dose of COVID-19 vaccine. For case growth model, the mean daily growth rate of new cases was 1%, and the mean effective vaccine coverage rate was 2.4 per 10 people (24% coverage rate).

**Table 2:** Summary of variables*

	Mean (SD)	Median (IQR)	N (%)
**Model I: human mobility**			
**Outcome: daily mobility change†**			
Retail and recreation in %	-13.2 (27.2)	-11.6 (-28.0, 2.8)	75 847
Groceries and pharmacy in %	8.3 (31.0)	5.0 (-7.3, 21.9)	75 837
Transit stations in %	-18.9 (28.2)	-20.1 (-36.5, -2.8)	75 782
Workplaces in %	-17.6 (19.7)	-16.1 (-28.0, -5.7)	75 791
**Covariates**			75 578
**Vaccine coverage (per 10 people)**			
No. of people received at least one dose	1.8 (2.7)	0.0 (0.0, 3.2)	
**Nonpharmaceutical interventions**			
Stringency index (0-100)	58.2 (19.4)	58.8 (45.4, 72.2)	
**Risk information (1 lag)**			
No. of new cases (10 000 people)	1.7 (4.9)	0.3 (0.0, 1.5)	
**Country characteristics**			
Hundreds of people per km^2^ of land	3.0 (10.3)	0.9 (0.3, 2.2)	
Population aged 65 y or above in %	11.5 (6.7)	9.9 (5.3, 18.1)	
GDP per capita in thousands international US$	25.1 (21.0)	18.9 (8.3, 35.9)	
Gini index (0-100)	37.8 (9.1)	35.9 (31.4, 41.9)	
Population that is female in %	50.0 (4.0)	50.5 (50.0, 51.2)	
Europe			24 855 (32.9)
North America			9124 (12.1)
South America			7038 (9.3)
Asia			22 715 (30.1)
Africa			9701 (12.8)
Oceania			2145 (2.8)
**Time factors**			
Spring			18 709 (24.8)
Summer			19 391 (25.7)
Autumn			18 786 (24.9)
Winter			18 692 (24.7)
**Model II: COVID-19 case growth**			
Total			39 405
**Outcome: case growth**			
Daily growth of new cases in %‡	1.0 (2.4)	0.0 (-3.0, 3.7)	
**Covariates**			
**Effective vaccine coverage**			
No. of people received at least one dose from 7 d and 6 mo ago (per ten people)	2.4 (2.1)	1.9 (0.5, 4.1)	
**Daily mobility change (14 lags)**			
Mobility index in %	-2.2 (23.1)	-3.3 (-15.5, 9.9)	
Retail and recreation in %	-4.3 (26.0)	-4.0 (-19.5, 10.9)	
Groceries and pharmacy in %	20.6 (31.7)	16.2 (3.0, 34.7)	
Transit stations in %	-10.7 (28.7)	-13.5 (-25.0, -3.8)	
Workplaces in %	-14.6 (19.5)	-14.0 (-25.0, -3.8)	
**Nonpharmaceutical interventions (14 lags)**			
Stringency index (0-100)	55.7 (56.0)	56.0 (44.4, 69.4)	
Facial covering: no policy			341 (0.9)
Facial covering: recommended			1369 (3.5)
Facial covering: required in some places			9201 (23.3)
Facial covering: required in all places			17 574 (44.6)
Facial covering: required in all places at all times			10 920 (27.7)
**Time factors**			
Delta period (11 May to 25 November 2021)			21 138 (53.6)
Omicron period (26 November 2021 to 6 February 2022)			7605 (19.3)
Spring			9321 (23.7)
Summer			9693 (24.6)
Autumn			9302 (23.6)
Winter			11 089 (28.1)

SD – standard deviation, GDP – gross domestic product

*Both models analysed 107 countries; standard errors were clustered at the country level. Mobility model used the full study periods (15 February 2020 – 6 February 2022). Case growth model used the post-vaccination periods. Four outcome variables in mobility model had different country-days; therefore, covariates in mobility model were calculated based on the smallest sample size, the transit stations regression.

†Mobility change was calculated as the percentage change in visits for a particular day relative to the normal (pre-pandemic) value for that day of the week.

‡Case growth was calculated based on the growth of seven-day moving average of new cases.

### Vaccine coverage positively associated with mobility, for all countries and by income group

Results obtained from the mobility model supported the VM hypothesis (**Table 3**). The all-country regression analyses revealed that when the vaccine coverage rate increased by 10 percentage points, the number of visitors to grocery stores, retail stores, transit stations, and workplaces increased by 4.28 (95% confidence intervals (CI) = 3.65, 4.92, *P* < 0.001), 3.59 (95% CI = 3.10, 4.09, *P* < 0.001), 2.72 (95% CI = 2.17, 3.27, *P* < 0.001), and 1.38 (95% CI = 1.06, 1.69, *P* < 0.001) percentage points, respectively. Our subgroup analyses revealed that for the same location, the magnitude of mobility increases was the greatest for LMI countries, followed by UMI and HI countries.

**Table 3:** Results from mobility model, for all countries and by income group*

Daily mobility change (%)	Retail and recreation		Grocery and pharmacy		Transit stations		Workplaces	
	Coef. (95% CI)	*P*>z	Coef. (95% CI)	*P*>z	Coef. (95% CI)	*P*>z	Coef. (95% CI)	*P*>z
**All countries**								
n of people vaccinated (per ten people)	3.59 (3.10, 4.09)	<0.001	4.28 (3.65, 4.92)	<0.001	2.72 (2.17, 3.27)	<0.001	1.38 (1.06, 1.69)	<0.001
Stringency index	-0.78 (-0.83, -0.72)	<0.001	-0.57 (-0.65, -0.49)	<0.001	-0.76 (-0.82, -0.70)	<0.001	-0.46 (-0.50, -0.41)	<0.001
n of new cases (per 10 000 people, 1 lag)	-0.46 (-0.65, -0.28)	<0.001	-0.24 (-0.42, -0.07)	0.006	-0.45 (-0.62, -0.29)	<0.001	-0.17 (-0.26, -0.07)	0.001
n (n of countries)	75 643 (107)		75 633 (107)		75 578 (107)		75 587 (107)	
**High income countries**								
n of people vaccinated (per ten people)	2.73 (2.25, 3.21)	<0.001	3.24 (2.69, 3.79)	<0.001	2.03 (1.43, 2.64)	<0.001	0.96 (0.63, 1.30)	<0.001
Stringency index	-0.72 (-0.78, -0.65)	<0.001	-0.33 (-0.40, -0.27)	<0.001	-0.63 (-0.70, -0.56)	<0.001	-0.43 (-0.47, -0.38)	<0.001
n of new cases (per 10 000 people, l lag)	-0.31 (-0.47, -0.16)	<0.001	-0.21 (-0.33, -0.10)	<0.001	-0.36 (-0.49, -0.22)	<0.001	-0.10 (-0.19, -0.02)	0.013
n (n of countries)	35 582 (50)		35 576 (50)		35,557 (50)		35 579 (50)	
**Upper-middle income countries**								
n of people vaccinated (per ten people)	4.93 (3.80, 6.06)	<0.001	5.98 (4.85, 7.11)	<0.001	4.05 (2.94, 5.16)	<0.001	2.07 (1.39, 2.76)	<0.001
Stringency index	-0.69 (-0.78, -0.60)	<0.001	-0.56 (-0.65, -0.46)	<0.001	-0.74 (-0.85, -0.64)	<0.001	-0.45 (-0.50, -0.39)	<0.001
n of new cases (per 10 000 people, l lag)	-0.65 (-1.04, -0.26)	0.001	0.14 (-0.24, 0.53)	0.458	-0.61 (-0.94, -0.27)	<0.001	-0.39 (-0.69, -0.08)	0.013
n (n of countries)	21 074 (30)		21 076 (30)		21 040 (30)		21 112 (30)	
**Lower-middle income countries**								
No. of people vaccinated (per ten people)	6.00 (4.41, 7.58)	<0.001	7.90 (5.31, 10.48)	<0.001	4.50 (2.73, 6.26)	<0.001	2.54 (1.33, 3.75)	<0.001
Stringency index	-0.89 (-1.00, -0.77)	<0.001	-0.90 (-1.03, -0.77)	<0.001	-0.93 (-1.08, -0.77)	<0.001	-0.50 (-0.63, -0.38)	<0.001
n of new cases (per 10 000 people, 1 lag)	-0.20 (-1.04, 0.64)	0.638	1.24 (0.19, 2.30)	0.021	0.52 (-0.54, 1.58)	0.334	0.27 (-0.44, 0.97)	0.455
n (n of countries)	18 987 (27)		18 981 (27)		18 981 (27)		18 896 (27)	

Coef – coefficient, CI – confidence interval

*Random-effects models were applied. Other covariates not shown in the table were: population density, the share of people aged 65 or over, GDP per capita, Gini index, % female population, and indicators for continent and season. Countries were categorized into three income groups based on the 2020 per capita gross national income. Standard errors were clustered at the country level.

### VM declined with vaccination stage, for all countries and by income group

**Figure 2:**
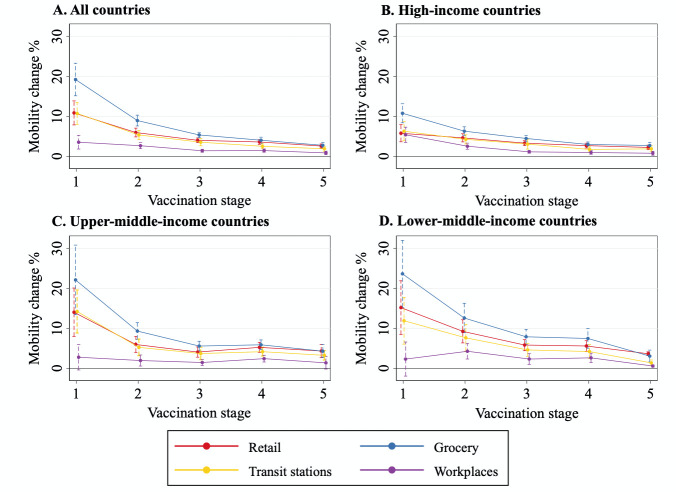
Association between mobility change and vaccine coverage at different stages of mass vaccination. This figure illustrates the nonlinear relationship between human mobility and vaccine coverage rate estimated by the random-effects model, after NPIs, risk information, country characteristics, and seasonality were controlled for. Vaccination stage was determined on the basis of the proportion of the population receiving at least one dose of COVID-19 vaccines: above 0 and ≤20% (stage 1), 20%-40% (stage 2), 40%-60% (stage 3), 60%-80% (stage 4), and ≥80% (stage 5); the reference stage is the pre-vaccination period. The vertical line represents the 95% confidence interval; the dot represents the estimated coefficient for the interactions between vaccine coverage and stage. The regression results are presented in Table S7, Appendix S7 in the **Online Supplementary Document****. Panel A.** Data for all 107 countries. **Panel B.** 50 HI countries. **Panel C.** 30 UMI countries. **Panel D.** 27 LMI countries.

Behavioural responses may change over time, leading to a nonlinear association between mobility and vaccine coverage. Therefore, we explored whether VM varied with the stage of vaccination. We categorised coverage into five stages (dummy variables): above 0 and <20% (stage 1), 20%-40% (stage 2), 40%-60% (stage 3), 60%-80% (stage 4), and ≥80% (stage 5). The reference group was no coverage, i.e., the pre-vaccination period. We added interactions between these five dummy variables (0/1) and vaccine coverage to the mobility model. The derived interaction terms are presented in **Figure 2** and Table S7 in Appendix S7 of the **Online Supplementary Document**.

For most categories of location, the increases in mobility were greatest at the initial stage of vaccination and became smaller as the coverage rate expanded. Consider, for example, the all-country sample (**Figure 2**, Panel A); compared with the pre-vaccination period, when the vaccine coverage rate increased by 10 percentage points, mobility in grocery stores (blue line) increased by 19.17 (95% CI = 15.11, 23.24), 8.92 (95% CI = 7.57, 10.27), 5.32 (95% CI = 4.66, 5.99), 4.01 (95% CI = 3.25, 4.76), and 2.73 (95% CI = 2.07, 3.39) percentage points at Stages 1, 2, 3, 4, and 5, respectively (*P* < 0.001).

### Negative association of vaccine coverage with case growth was stronger after adjustment for human mobility

**Figure 3:**
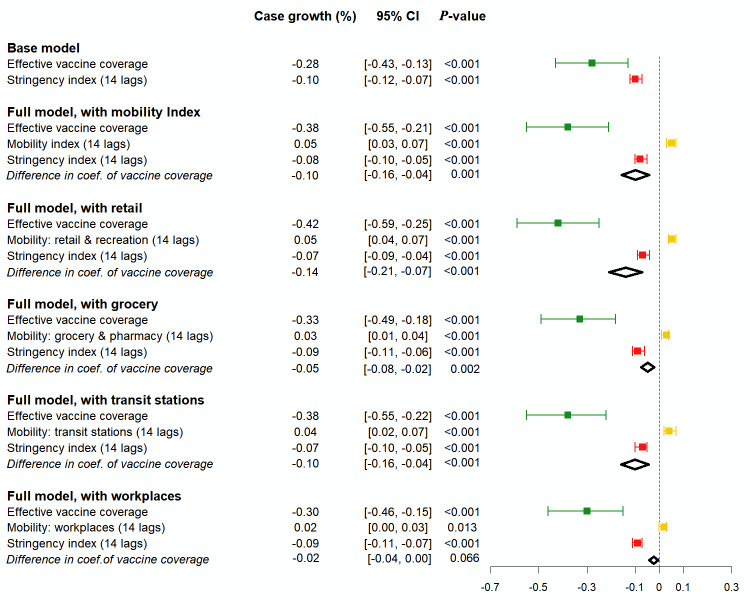
Association between case growth and vaccine coverage, with and without adjustment for human mobility. The negative association between case growth and vaccine coverage is illustrated by the green line, where the dot in the middle denotes the estimated coefficient of effective vaccine coverage. The full models included mobility variables while the base model did not. The case growth was calculated as the daily growth rate of the 7-day moving average of new cases. Effective vaccine coverage was the cumulative number of people who received at least one dose of COVID-19 vaccines per 10 people from seven days and six months ago. The sample was a longitudinal data set comprising data collected from 107 countries with 39 450 country-days. Only the postvaccination period was used for analysis. The included common covariates not shown in the figure were face-covering policies, indicators for season, Delta period, and Omicron period. Fixed-effects models were applied; standard errors were clustered at the country level.

Results obtained from case growth model are displayed in **Figure 3**. The base model, which excluded mobility, indicated that when the effective vaccine coverage rate increased by 10 percentage points, the daily case growth rate decreased by 0.28 percentage points (95% CI = -0.43, -0.13, *P* < 0.001). However, the full models indicated stronger protection effects. Specifically, when the effective vaccine coverage rate increased by 10 percentage points, the daily case growth rate decreased by 0.38 (95% CI = -0.55, -0.21, *P* < 0.001), 0.42 (95% CI = -0.59, -0.25, *P* < 0.001), 0.33 (95% CI = -0.49, -0.18, *P* < 0.001), 0.38 (95% CI = -0.55, -0.22, *P* < 0.001), and 0.3 (95% CI = -0.46, -0.15, *P* < 0.001) percentage points, respectively, when the mobility index and mobility in retail stores, grocery stores, transit stations, and workplaces were controlled for.

The difference in vaccine coverage coefficient between the base and full models is indicated in **Figure 3**. For the full model with the mobility index, the coefficient difference was -0.1 ( 95% CI = -0.16, -0.04, *P* = 0.001). Similarly, after adjustment for mobility in retail stores, grocery stores, and transit stations, the difference in coefficient of vaccine coverage was -0.14 (95% CI = -0.21, -0.07, *P* < 0.001), -0.05 (95% CI -0.08, -0.02; *P* = 0.002), and -0.1 (95% CI = -0.16, -0.04; *P* < 0.001) percentage points, respectively. This means that VM in retail stores, grocery stores, and transit stations attenuated vaccine effectiveness in curbing case growth by 33% (0.14/0.42), 15% (0.05/0.33), and 26% (0.1/0.38), respectively. Only workplace mobility exhibited no mediating role in the vaccine-infection relationship. These results indicate that the effectiveness of COVID-19 vaccination could be underestimated if VM is not considered.

### Results from robustness checks and false-positive corrections

The main findings from robustness checks appeared to be consistent with those from the primary models. An exception was that, when we included the number of different vaccines in the mobility model, which reduced the sample from 107 to 38 countries, mobility in grocery stores did not appear to attenuate vaccine effectiveness (Table S6.4, Appendix S6 in the **Online Supplementary Document**). To reduce the likelihood of false rejections, we adjusted the *P*-values and presented the results in Appendix S8 in the **Online Supplementary Document**. The application of false-positive corrections did not change the study results.

## DISCUSSION

We examined whether COVID-19 vaccine coverage increased human mobility and investigated the extent to which VM attenuated the effectiveness of COVID-19 vaccines. All-country regression analyses indicated that a 10-percentage-point increase in vaccine coverage was independently associated with a 1.38-4.28 percentage-point increase in mobility. Moreover, across income groups, grocery stores were consistently associated with the greatest VM, followed by retail stores, transit stations, and workplaces. VM appeared to be greater for lower-income countries and at earlier stages of mass vaccination. We observed that VM attenuated the vaccine effectiveness in controlling case growth by 33.4% in retail stores, 26.4% in transit stations, and 15.4% in grocery stores. Our findings of the unintended consequences of VM will be instrumental to mitigation policy in the postvaccination era.

### COVID-19 vaccine coverage associated with increased human mobility

The positive association of vaccine coverage with human mobility supports the view that COVID-19 vaccination may have led to the Peltzman effect [2,4]. A comprehensive review identified four factors required to trigger risk compensation behaviour: visibility, motivation, control, and effect [32]. All four factors are relevant for our study because people are aware of being vaccinated (visibility), want to return to pre-pandemic life (motivation), can easily increase mobility after the lifting of lockdown-regulations (control), and have learnt the benefits of COVID-19 vaccination through public campaigns (effect) [2,4]. This finding is consistent with those of previous surveys, in which respondents reported that they would reduce their practice of COVID-19 protective behaviours if they were determined to have antibodies to SARS-CoV-2 [33-35].

### VM varied with location, income group, and vaccination stage

Across the four mobility categories, grocery stores were associated with the greatest VM. This category of location includes supermarkets, food warehouses, farmers’ markets, specialty food shops, and pharmacies [19], which all serve basic human needs. The high VM in these locations suggests that people prioritised essential trips after vaccination, especially in LMI countries. Compared with the other locations, workplaces had the lowest VM. This seems plausible because mobility to workplaces was subject to environmental factors, such as occupation and employment status. The pandemic has caused job losses and a shift towards part-time employment [36]. In this context, those being adversely affected would be less likely to visit workplaces even after vaccination. Moreover, across income groups, VM was lowest in HI countries. This may be because work from home has become a new norm in the developed world [37].

Results indicated that VM was smaller for HI countries. To explore possible explanations, we looked at nonmonetary measures of socioeconomic development status (SDS) across income groups. The selected SDS variables and descriptive statistics are provided in Table S9.1 and Table S9.2, Appendix S9 in the **Online Supplementary Document**. Compared with UMI and LMI countries, HI countries had higher SDS, characterized by a higher school enrolment ratio, higher trust in science and people in the neighbourhood, a considerably smaller share of agricultural and self-employed workers, and a substantially larger share of the population using the internet. In further assessing whether SDS moderated the relationship between vaccine coverage and mobility, we added SDS and their interactions with vaccine coverage to the mobility model. The regression results are presented in Table S9.3 and the marginal effects are in Table S9.4 in the **Online Supplementary document**. The results showed that VM decreased with (an increase in) school enrolment ratio, trust in science and people, and the share of internet users. However, VM increased with the share of agricultural and self-employed workers in a country. These findings consistently revealed that VM was smaller in countries with higher SDS, which helped explain why mobility response was smaller in higher-income countries. In literature, good education was found to mitigate risky health behaviour [38]. Trust is a key element of social capital, which may contribute to collective preventive behaviour in the absence of mobility control [39]. Furthermore, agriculture sector often required human presence to operate. The widespread internet use may facilitate a decrease in mobility, such as substituting online shopping for visiting physical stores.

We observed a considerable surge in mobility at the initial stage of the vaccine rollout. This is particularly concerning because high mobility combined with low vaccine coverage rates could pose a high risk of infection. Studies conducted in Israel and the United Kingdom have reported a spike in COVID-19 infection shortly after individuals’ first injections [40,41]. Our findings provide a potential explanation for these observations and support the concern that people may be overly complacent before population immunity against COVID-19 has reached a sufficiently high level [42].

### VM attenuated, but not completely countered, vaccine effectiveness

We found that mobility mediated or weakened the negative relationship between vaccination and case growth. Compared with the mean daily case growth rate of 1% (**Table 2**), the degree of underestimation (between 0.05 and 0.14 percentage points) caused by the omission of the mobility variable in the base model was not negligible. These results suggest that VM could offset vaccine effectiveness partly but not completely. People still benefit from a decreased risk of contracting COVID-19 after vaccination; however, the benefit is less than the expected level owing to the compensatory mobility increase.

We observed that mobility in the retail stores category appeared to attenuate the vaccine effectiveness to the greatest degree (by approximately 33%). This category comprises recreational places such as restaurants, cafés, shopping centres, theme parks, museums, libraries, and cinemas [19]. This finding is consistent with that of a simulation study, which reported that reopening restaurants, gyms, hotels, and cafés produced the largest predicted increases in infections [29]. Mobility in these places is the most risky, probably because people stay there longer, are more likely to not wear masks, or are less able to control who they come into contact with or maintain some physical distance from [29].

Previously, we discussed that mobility in grocery stores increased most considerably after vaccination; however, mobility in such stores attenuated vaccine effectiveness (15%) less than mobility in retail stores (33%) and transit stations (26%) did. Notably, mobility in workplaces did not seem to affect vaccine effectiveness, and this may be due to the implementation of precautionary measures in workplaces.

### Strategies to counter the potential unintended consequences of VM

On the basis of our study findings, we suggest several strategies that could be considered for mitigating the unintended consequences of VM. First, strengthening the management of short-term mobility responses after vaccination is vital. Because mobility was observed to surge most dramatically at the initial stage of vaccine rollout, relevant authorities must educate people about the timing of immunity generated by COVID-19 vaccines. Research revealed that only 44% of US adults know that strong protection did not occur one to two weeks after the second dose [43]. Campaigns for rectifying the “false sense of increased security” [2,4] and misperceptions about immediate protection after vaccination [15,44] may help reduce initial increases in mobility. Second, the sequence through which venues are reopened is crucial for the control of virus transmission. We observed that mobility in workplaces and grocery-type places had relatively low effects on vaccine effectiveness; therefore, mobility in such places may be prioritised. Authorities should reconsider whether restricting mobility in such places postvaccination is warranted. Finally, lower-income countries were observed to have higher VM. Coupled with the fact that those countries had a slow pace of vaccine coverage, there could be larger windows for virus transmission when compared with those in HI countries. Therefore, LMI countries should implement rapid vaccine rollout processes to ensure the achievement of certain coverage rates (e.g., 20% coverage in Stage 1).

### Limitations

This study has some limitations. First, data from Google Community Mobility Reports are confined to people who activated location history in the Google Maps application; therefore, children and older adults may be underrepresented. Second, we could not obtain individual-level data and the corresponding socioeconomic characteristics; thus, we could not analyse heterogeneity in behavioural responses across population strata. Third, although we included a wide range of control variables, confounding factors could still influence the mobility outcome. We attempted to address this potential problem by instrumenting vaccine coverage in our robustness checks, but additional studies may employ alternative approaches to address the problem. Fourth, our estimated VM may also be partly explained by the Bystander effect, which states that the presence of other bystanders reduces an individual’s feelings of personal responsibility [45]. After a vaccine rollout, simply observing other people get vaccinated might prompt unvaccinated individuals to reduce their social and physical distancing behaviours [4]. We could not detect mobility differences between vaccinated and unvaccinated individuals in the Google data. Consequently, VM may have captured the spillover effect of vaccination on mobility. Fifth, this study was not able to validate or harmonize data reported by individual countries. Pooling cross-country data would oversimplify the complexity of data. To the extent that the time-invariant country-specific factors did not fully capture variation in data collection, this study would suffer from measurement errors. Sixth, obtaining data from over 100 countries has been a challenge. A lack of data has limited our research design. Although we have performed a series of robustness checks on the association between vaccine coverage, mobility and case growth, this study was not a randomized trial, and our analysis was not sufficient to make causal inferences. Finally, we focused on only mobility responses; future research may investigate other behavioural adjustments and their consequences on vaccine effectiveness.

## CONCLUSION

Using longitudinal data from 107 countries over two years, we investigated behavioural responses to COVID-19 vaccination. We observed that vaccine coverage was positively associated with human mobility, a finding that supports the Peltzman effect. Such increases in mobility were greater in lower-income countries and in earlier stages of vaccine rollouts. Crucially, VM in retail and recreational places attenuated vaccine effectiveness by approximately one-third. By contrast, the unintended consequences of VM were smaller in grocery stores and pharmacies and were not statistically significant in workplaces. Overall, the phenomenon of VM has prevented mass vaccination from providing maximum benefits; nevertheless, we suggest strategies for countering the effects of VM. Since the vaccine rollout, governments have faced the dual challenge of boosting the vaccine rate while encouraging people to continue following protective measures. Our findings are instrumental for the control of the COVID-19 pandemic postvaccination.

## Additional material


Online Supplementary Document
