# Supplementary material for: Human mobility increased with vaccine coverage and attenuated the protection of COVID-19 vaccination: A longitudinal study of 107 countries
Source: J Glob Health. 2023 Apr 7;13:06009. doi: 10.7189/jogh.13.06009 (PMC10078856; doi:10.7189/jogh.13.06009)
Supplement: Online Supplementary Document [file jogh-13-06009-s001.pdf]

SUPPLEMENTARY MATERIALS

Human mobility increased with vaccine coverage and attenuated the protection of COVID-19 vaccination: A longitudinal study of 107 countries

Appendix S1 Flow chart of sample selection 2

Figure S1 Flow diagram of sample selection 2

Appendix S2 List of countries and control variables 3

Table S2 107 countries analysed and country characteristics 3

Appendix S3 Empirical models for human mobility and case growth 7

Appendix S4 Gelbach decomposition method 9

Appendix S5 Robustness checks for mobility model 10

Table S5.1 Results from mobility model, assuming country fixed effects 11

Table S5.2 Results from mobility model, using the 7-day moving average for mobility outcome 12

Table S5.3 Results from mobility model: using the lagged vaccine coverage as key predictor 13

Table S5.4 Results from mobility model: using instrumental variables for vaccine coverage 14

Table S5.5 Results from mobility model: adding the number of boosters and interaction with vaccine coverage 15

Appendix S6 Robustness check for case growth model 16

Table S6.1 Results from case growth model, using the reproduction rate as outcome 18

Table S6.2 COVID-19 vaccines granted Emergency Use Listing by WHO, FDA, and EMA 19

Table S6.3 Cumulative vaccine doses in 38 countries (including 27 EU countries) as of 6 February 2022, by manufacturer (unit: million) 20

Table S6.4 Results from case growth model: adding number of different COVID-19 vaccines 21

Appendix S7 Mobility model: adding interactions between vaccine coverage rate and stage of vaccination 23

Table S7 Results from mobility model: adding interactions between vaccine coverage rate and stage of vaccination 23

Appendix S8 Applying false-positive corrections to mobility and case growth models 25

Table S8.1 Results of applying false-positive correction to mobility model presented in Table 3 26

Table S8.2 Results of applying false-positive correction to case growth model presented in Figure 3 27

Appendix S9 Mobility model: adding country socioeconomic development status (SDS) as moderators 28

Table S9.1 Summary of variables for country socioeconomic development status 30

Table S9.2 Mean value for country socioeconomic development status (SDS), by income group 31

Table S9.3 Results from mobility model: adding SDS and interaction with vaccine coverage 32

Table S9.4 Average marginal effects of vaccine coverage on mobility, at different values of SDS 33

Appendix S1 Flow chart of sample selection

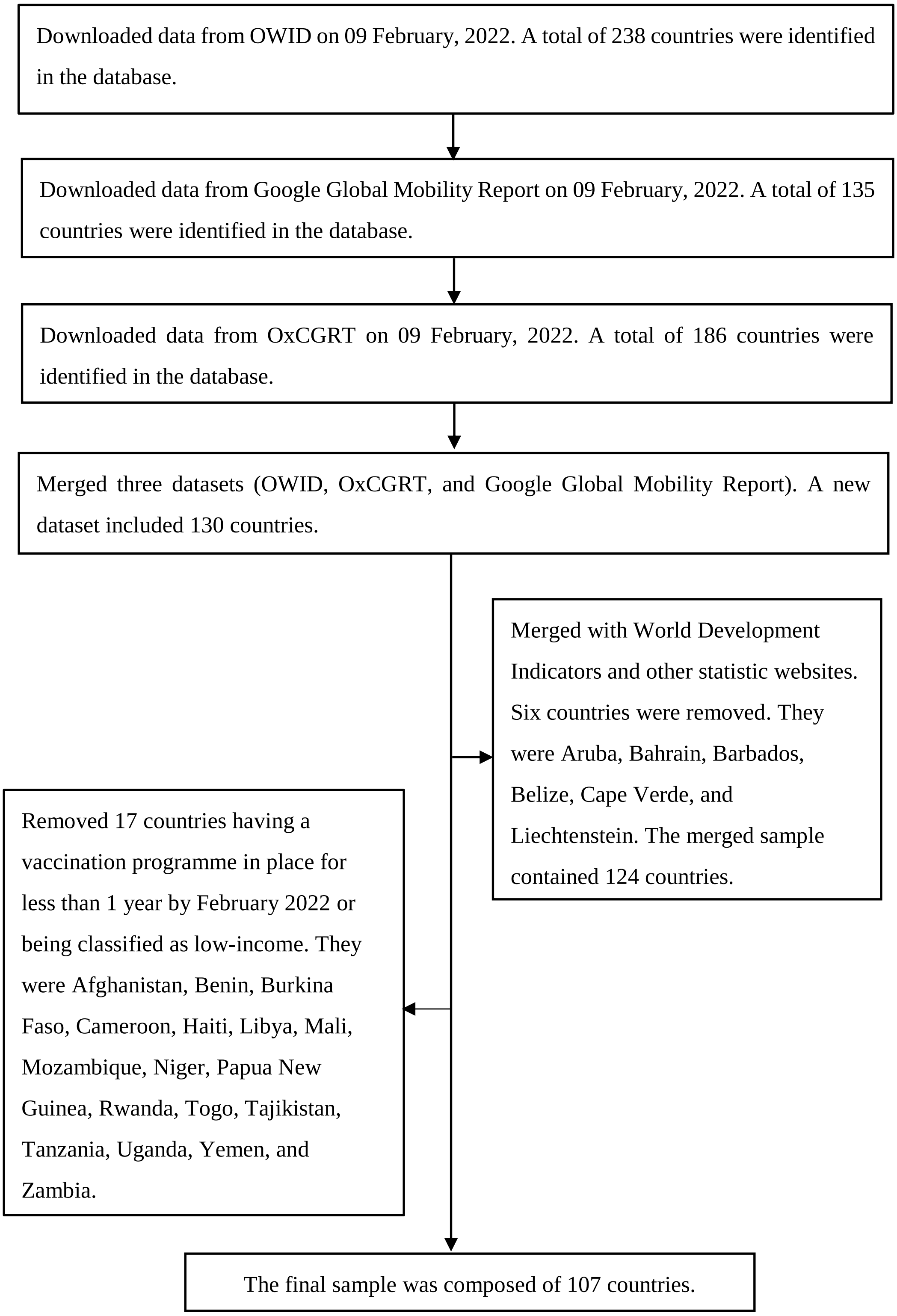

Figure S1 Flow diagram of sample selection

Appendix S2 List of countries and control variables

Control variables

Country characteristics

We controlled for time-invariant country characteristics that may have affected human mobility and case growth. The following country variables were used in this study: population density (hundreds of people per square kilometres of land), proportion of the population aged ≥65 years (%), per capita gross domestic product at purchasing power parity (measured in thousands of constant 2011 international dollars), the Gini index (0–100), female proportion (%), and continent indicator for each country (Europe, North America, South America, Asia, Africa, and Oceania). The data were downloaded from the World Development Indicators database published by the World Bank (https://databank.worldbank.org/source/world-development-indicators). Data for the prepandemic year 2019 were used.

Time factors

To control for seasonality, we included seasonal indicators in both mobility and case growth models. Because seasons differ between the northern and southern hemispheres, we manually reviewed the location of each country when constructing the variables. Moreover, to capture differences in transmissibility, the case growth model included indicators for periods when the Delta and Omicron variants were declared by the World Health Organization to be Variants of Concern (Delta: 11 May–25 November 2021, Omicron: 26 November 2021 onwards).

Table S2 provides the list of sample countries and the corresponding country characteristics.

Table S2 107 countries analysed and country characteristics

Country	Income level[a]	Hundreds of people per km^2 of land	Population aged 65 years or above (%)	GDP per capita (thousands of 2011 international $)	Gini index (0-100)	Female population (%)
Angola	LMI	0.2	2.2	5.8	51.3	51
Argentina	UMI	0.2	11.2	18.9	42.9	51.1
Australia	HI	<0.1	15.9	44.6	34.4	50.2
Austria	HI	1.1	19.1	45.4	30.8	51
Bahamas	HI	0.4	7.5	27.7	57	51
Bangladesh	LMI	12.4	5.2	3.5	32.4	49.6
Belarus	UMI	0.5	15.2	17.2	25.3	53.5
Belgium	HI	3.8	19	42.7	27.2	50.7
Bolivia	LMI	0.1	7.3	6.9	41.6	49.9
Bosnia and Herzegovina	UMI	0.6	17.2	11.7	33	50.9
Brazil	UMI	0.3	9.3	14.1	53.4	50.9
Bulgaria	UMI	0.6	21.3	18.6	41.3	51.4
Botswana	UMI	<0.1	4.4	15.8	53.3	50.6

Country	Income level	Hundreds of people per km^2 of land	Population aged 65 years or above (%)	GDP per capita (thousands of 2011 international $)	Gini index (0-100)	Female population (%)
Cambodia	LMI	0.9	4.7	3.6	36.6	51.2
Canada	HI	<0.1	17.6	44	33.3	50.4
Chile	HI	0.3	11.9	22.8	44.4	50.5
Colombia	UMI	0.4	8.8	13.3	51.3	50.8
Costa Rica	UMI	1.0	9.9	15.5	48.2	50
Cote d'Ivoire	LMI	0.8	2.9	3.6	41.5	49.3
Croatia	HI	0.7	20.9	22.7	29.7	51.8
Czech	HI	1.4	19.8	32.6	25	50.8
Denmark	HI	1.4	20	46.7	28.2	50.3
Dominican Republic	UMI	2.2	7.3	14.6	41.9	50.2
Ecuador	UMI	0.7	7.4	10.6	45.7	50
Egypt	LMI	1.0	5.3	10.6	31.5	49.4
El Salvador	LMI	3.1	8.5	7.3	38.8	53
Estonia	HI	0.3	20	29.5	30.3	53.1
Fiji	UMI	0.5	5.6	8.7	36.7	49.2
Finland	HI	0.2	22.1	40.6	27.3	50.7
France	HI	1.2	20.4	38.6	32.4	50.8
Gabon	UMI	0.1	3.5	16.6	38	48.6
Georgia	UMI	0.7	15.1	9.7	35.9	52.3
Germany	HI	2.4	21.6	45.2	31.9	50.8
Ghana	LMI	1.3	3.1	4.2	43.5	50.2
Greece	HI	0.8	21.9	24.6	32.9	50.8
Guatemala	UMI	1.5	4.9	7.4	48.3	50.8
Honduras	LMI	0.9	4.8	4.5	48.2	50.1
Hong Kong	HI	71	17.5	56.1	54	54
Hungary	HI	1.1	19.7	26.8	29.6	52.4
India	LMI	4.5	6.4	6.4	47.9	48.2
Indonesia	LMI	1.5	6.1	11.2	38.2	49.7
Iraq	UMI	0.9	3.4	15.7	29.5	49.4
Ireland	HI	0.7	14.2	67.3	31.4	50.4
Israel	HI	4.1	12.2	33.1	39	50.3
Italy	HI	2.1	23	35.2	35.9	51.3
Jamaica	UMI	2.7	8.9	8.2	45.5	50.2
Japan	HI	3.5	28	39	32.9	51.2
Jordan	UMI	1.1	3.9	8.3	33.7	49.4
Kazakhstan	UMI	0.1	7.7	24.1	27.8	51.6

Country	Income level	Hundreds of people per km^2 of land	Population aged 65 years or above (%)	GDP per capita (thousands of 2011 international $)	Gini index (0-100)	Female population (%)
Kenya	LMI	0.9	2.4	3	40.8	50.3
Kuwait	HI	2.3	2.8	65.5	80.9	42.6
Kyrgyzstan	LMI	0.3	4.6	3.4	29.7	50.4
Laos	LMI	0.3	4.2	6.4	38.8	50.1
Latvia	HI	0.3	20.3	25.1	35.1	54.1
Lebanon	UMI	6.7	7.3	13.4	31.8	49.8
Lithuania	HI	0.4	20.2	29.5	35.7	53.9
Luxembourg	HI	2.5	14.3	94.3	35.4	49.7
Malaysia	UMI	1.0	6.9	26.8	41.1	48.4
Malta	HI	15.1	20.8	36.5	28.7	49.8
Mauritius	HI	6.2	12	20.3	36.8	50.5
Mexico	UMI	0.6	7.4	17.3	45.4	50.2
Moldova	UMI	0.9	12	5.2	25.7	52
Mongolia	LMI	<0.1	4.2	11.8	32.7	50.5
Morocco	LMI	0.8	7.3	7.5	39.5	50.5
Myanmar	LMI	0.8	6	5.6	30.7	51.2
Namibia	UMI	<0.1	3.6	9.5	59.1	51.4
Nepal	LMI	2.0	5.8	2.4	32.8	51.5
Netherlands	HI	5.1	19.6	48.5	28.1	50.2
New Zealand	HI	0.2	16	36.1	41.9	50.8
Nicaragua	LMI	0.5	5.5	5.3	46.2	50.7
Nigeria	LMI	2.2	2.7	5.3	35.1	49.3
Norway	HI	0.1	17.3	64.8	27.6	49.5
Oman	HI	0.2	2.4	38	30.7	34.2
Pakistan	LMI	2.8	4.3	5	31.6	48.6
Panama	HI	0.6	8.3	22.3	49.8	49.9
Paraguay	UMI	0.2	6.6	8.8	45.7	49.3
Peru	UMI	0.2	8.4	12.2	41.5	50.1
Philippines	LMI	3.6	5.3	7.6	37.8	49.7
Poland	HI	1.2	18.1	27.2	30.2	51.7
Portugal	HI	1.1	22.4	27.9	33.5	52.7
Qatar	HI	2.4	1.5	116.9	41.1	24.9
Romania	HI	0.8	18.8	23.3	35.8	51.6
Russia	UMI	0.1	15.1	24.8	37.5	53.5
Saudi Arabia	HI	0.2	3.4	49	45.9	42.9

Country	Income level	Hundreds of people per km^2 of land	Population aged 65 years or above (%)	GDP per capita (thousands of 2011 international $)	Gini index (0-100)	Female population (%)
Senegal	LMI	0.8	3.1	2.5	40.3	50.9
Serbia	UMI	0.8	18.7	14	30.4	51.1
Singapore	HI	79.5	12.4	85.5	37.5	50.6
Slovakia	HI	1.1	16.2	30.2	25	51.4
Slovenia	HI	1.0	20.2	31.4	24.6	50.3
South Africa	UMI	0.5	5.4	12.3	63	50.9
South Korea	HI	5.3	15.1	35.9	31.4	50
Spain	HI	0.9	19.6	34.3	34.7	51
Sri Lanka	LMI	3.5	10.8	11.7	39.3	51.9
Sweden	HI	0.2	20.2	46.9	30	50
Switzerland	HI	2.2	18.8	57.4	33.1	50.5
Taiwan	HI	6.7	16	32.8	31.5	50.1
Thailand	UMI	1.4	12.4	16.3	34.9	51.2
Trinidad and Tobago	HI	2.7	11.1	28.8	40.3	50.7
Turkey	UMI	1.1	8.7	25.1	41.9	50.7
Ukraine	LMI	0.8	16.7	7.9	26.6	53.8
United Arab Emirates	HI	1.4	1.2	67.3	26	27.6
United Kingdom	HI	2.7	18.5	39.8	35.1	50.7
United States	HI	0.4	16.2	54.2	41.4	50.5
Uruguay	HI	0.2	14.9	20.6	39.7	51.7
Venezuela	HI	0.3	7.6	16.7	44.8	50.3
Vietnam	LMI	3.1	7.6	6.2	35.7	50.5
Zimbabwe	LMI	0.4	3	1.9	50.3	51.3

Note: HI: High income. UMI: upper-middle-income. LMI: lower-middle-income. GDP: gross domestic product.

[a.] We used the 2022 World Bank classification, which defined income groups on the basis of 2020 per capita gross national income (GNI). HI economies referred to those with a GNI per capita of $12,695 or more. UMI economies were those with a GNI per capita between $4,096 and $12,695, and LMI economies were those with a GNI per capita between $1,046 and $4,095.

Appendix S3 Empirical models for human mobility and case growth

Model I: Human mobility

Individuals may increase their mobility after vaccination against COVID-19. Therefore, we parameterised mobility as follows:

$$mobility_{i,t}^{k} = \beta_1^k + \beta_2^k vaccine_{i,t} + \beta_3^k stringnecy_{i,t} + \beta_4^k newcases_{i,t-1} + C_i' B_5^k + S_t' B_6^k + v_i + \varepsilon_{i,t} \quad \text{(S3.1)}$$

where $mobility_{it}^{k}$ denotes the percentage change (relative to the baseline value) in the number of visitors to location k for country i at time t, $vaccine_{i,t}$ denotes the vaccine coverage, $stringnecy_{i,t}$ denotes the stringency index, and $newcases_{i,t-1}$ denotes the lagged number of new cases. VM was estimated by β_2^k, the change in mobility for one more person vaccinated against COVID-19 per 10 people in the population. C_i' and S_t' are the row vector for country characteristics and season indicators, respectively. Additionally, v_i represents unobservable time-invariant country-specific factors associated with mobility. Finally, ε_{it} is the usual idiosyncratic error.

Equation (S3.1) was estimated separately for the four categories of mobility (k = 1, …,4). According to the Hausman test [1], a random-effects model was preferable for some regression analyses, but a fixed-effects model was preferable for others. For consistency, we used a random-effects model as our primary model. The Breusch and Pagan Lagrange multiplier test [2] for random effects rejected our null hypothesis that the variance of v_i would be 0 (P < 0.001) for all regression analyses, supporting the inclusion of random effects. We present the results of our fixed-effects regressions in Table S5.1, Appendix S5; we observed that both models generated similar results. Furthermore, standard errors were clustered at the country level to account for correlations of mobility data within countries over time.

Model II: COVID-19 case growth and Gelbach decomposition

Vaccine coverage and mobility were key predictors in case growth model, which can be expressed as follows:

$$casegrowth_{i,t} = \gamma_1 + \gamma_2(vaccine_{i,t-7} - vaccine_{i,t-180}) + \gamma_3 mobility_{i,t-14} + \gamma_4 stringnecy_{i,t-14} + F_{i,t-14}'\Gamma_5 + C_i'\Gamma_6 + Z_t'\Gamma_7 + f_i + e_{i,t} \quad \text{(S3.2)}$$

where $casegrowth_{i,t}$ denotes the growth of new cases in country i at time t, as defined in Equation (1) in the main text. Moreover, $vaccination_{i,t-7} - vaccination_{i,t-180}$ denotes the effective coverage rate, calculated as the cumulative number of people who received at least one dose of COVD-19 vaccines per ten people from 7 days and 6 months ago. $F_{i,t-14}'$ and C_i' represent row vectors for face-covering policies and country characteristics, respectively. Z_t' represents indicators for seasons and Delta and Omicron periods. Mobility, the stringency index, and face-covering policies were all lagged by 14 days to account for incubation after contact and possible delays in case detection. Finally, f_i denotes time-invariant unobservable country effects. e_{it} denotes the idiosyncratic error.

Our objective was to evaluate changes in case growth within countries over time; furthermore, the Hausman test indicated that a fixed-effects model would be preferable to a random-effects model. Consequently, we treated f_i as fixed and thus could not estimate time-invariant country characteristics from Equation (S3.2). In addition, because the four

mobility variables (retail and recreation, grocery stores and pharmacies, transit stations, and workplaces) were highly correlated (correlation coefficient: 0.6−0.9, $P < 0.001$), we included them in the model individually. Standard errors were clustered at the country level to correct for serial correlations.

We examined whether VM would reduce the effectiveness of vaccines against COVID-19 infection. In estimating the indirect effect of mobility through vaccination, we adopted the decomposition method proposed by Gelbach [3]. Specifically, we compared the value of the vaccine coverage coefficient in Equation (S3.2) derived when mobility was excluded from the model (base model) with that derived when mobility was included in the model (full model).

References:

1. Hausman JA, Taylor WE. Panel data and unobservable individual effects. Econometrica 1981;49:1377-98.

2. Breusch TS, Pagan AR. The Lagrange multiplier test and its applications to model specification in econometrics. The Review of Economic Studies. 1980;47:239-53.

3. Gelbach JB. When do covariates matter? And which ones, and how much. Journal of Labour Economics. 2016;34:509–43.

Appendix S4 Gelbach decomposition method

By using least-squares identity, Gelbach (2016) [1] demonstrated that the difference in coefficient between the base and full models was equivalent to the omitted variables bias. Consider a population regression model (Gelbach, p.518)

$$Y = X_1\beta_1 + X_2\beta_2 + \varepsilon \qquad \text{(S4.1)}$$

and define $\beta = [\beta_1', \beta_2']'$, $X = [X_1, X_2]$. We call this *full model*. The ordinary least squares (OLS) estimator is given by $\hat{\beta} = (X'X)^{-1}X'Y$. Denote the first and second component of $\hat{\beta}$ as $\hat{\beta}_1^{full}$ and $\hat{\beta}_2$, respectively. Under usual conditions, $\hat{\beta}_1^{full}$ is a consistent estimator for β_1. Now suppose X_2 is omitted from the full model, and the base model is written as

$$Y = X_1\beta_1^{base} + \epsilon, \qquad \text{(S4.2)}$$

where the OLS estimator is $\hat{\beta}_1^{base} = (X_1'X_1)^{-1}X_1'Y$. Its probability limit is

$$plim\hat{\beta}_1^{base} = \beta_1 + plim(X_1'X_1)^{-1}X_1'X_2\beta_2, \qquad \text{(S4.3)}$$

$$\text{or } \beta_1^{base} = \beta_1 + \Gamma\beta_2 = \beta_1 + \delta, \qquad \text{(S4.4)}$$

where Γ is the matrix of coefficients from regressing X_2 on X_1, and δ represents the difference in the population coefficients between the base and full models, or the population omitted variables bias resulted from excluding X_2 when estimating β_1. Note that δ is non-zero when $E[X_1X_2] \neq 0$.

Gelbach (p. 521) further pointed out that since $plim\hat{\beta}_1^{base} = \beta_1 + \delta$, and because β_1 is consistent for $\hat{\beta}_1^{full}$, δ can be estimated as $\hat{\delta} \equiv \hat{\beta}_1^{base} - \hat{\beta}_1^{full}$, which is just empirically comparing the base and full models. To prove that $\hat{\delta}$ identically equals the sample omitted variables bias formula, $(X_1'X_1)^{-1}X_1'X_2\hat{\beta}_2$, let's express (S4.1) in the sample analogue:

$$\hat{Y} = X_1\hat{\beta}_1^{full} + X_2\hat{\beta}_2 + \hat{\varepsilon}, \qquad \text{(S4.5)}$$

where $\hat{\varepsilon}$ is the residuals. Pre-multiplying both sides by $(X_1'X_1)^{-1}X_1'$ generates $\hat{\beta}_1^{base} = \hat{\beta}_1^{full} + (X_1'X_1)^{-1}X_1'X_2\hat{\beta}_2$. Therefore, $\hat{\delta} \equiv \hat{\beta}_1^{base} - \hat{\beta}_1^{full}$=$(X_1'X_1)^{-1}X_1'X_2\hat{\beta}_2$.

In our case growth model, because fixed-effects were assumed, we first demeaned (centred) all covariates, and then estimated the model by usual OLS linear regressions before applying Gelbach formula.

Reference:

1. Gelbach JB. When do covariates matter? And which ones, and how much. Journal of Labour Economics. 2016;34:509–43.

Appendix S5 Robustness checks for mobility model

For robustness checks of mobility model, we used five alternative specifications. First, we applied the fixed-effects instead of random-effects regressions (Table S5.1). Second, we replaced the mobility outcome with its 7-day moving average (Table S5.2). Third, the key predictor, vaccine coverage was lagged by 7 days to allow for a delay in behaviour response (Table S5.3). Fourth, we used an instrumental variable approach to re-estimate the mobility model to tackle the potential existence of unobserved time-variant confounders. In this model, vaccine coverage was instrumented using the 2019 government effectiveness score obtained from Worldwide Governance Indicators [1,2] and an ordinal variable for COVID-19 vaccine availability obtained from the Oxford COVID-19 Government Response Tracker (OxCGRT) [3]. The government effectiveness score ranged from -2.5 to 2.5, with a higher value indicating better governance. The vaccine availability had three values: 0 refers to "no categories are receiving vaccines", 1 refers to "vaccines are available to some categories", and 2 refers to "vaccines are available to anyone over the age of 16 years". We selected these instrumental variables because they were highly correlated with the level of vaccine coverage but were unlikely to be correlated with mobility. The instrumental variable estimation resulted in a slightly increased magnitude of the positive coefficient of mobility, but the main conclusions remained robust (Table S5.4).

Finally, we investigated whether booster doses increased mobility and changed the association between mobility and vaccine coverage. A variable called "total boosters" was retrieved from Our World in Data [4]. The variable recorded the total number of COVID-19 booster doses administered each day per country. Boosters are only available for fully vaccinated people, namely, those who received all doses prescribed by the initial vaccination protocol. Therefore, we divided the cumulative number of boosters by the cumulative number of fully vaccinated people. The ratio was multiplied by 10 for the convenience of interpretation. The variable was then included in the mobility model and interacted with the vaccine coverage. The results are presented in Table S5.5. The average marginal effects showed that the number of boosters was not associated with human mobility in any location category. For example, the third to last row in the table showed that, the number of visitors to retail and recreation increased by 0.27 percentage point when the number of boosters (per ten fully vaccinated people) increased by one; however, the association was not statistically significant (95% confidence interval -3.16 to 3.69; P= 0.879). Overall, the inclusion of booster variables did not change the key finding that COVID-19 vaccine coverage was associated with increased human mobility.

References:

1. Kaufmann D, Kraay A, Mastruzzi M. The Worldwide Governance Indicators: methodology and analytical Issues. Washington DC: The World Bank, 2010.

2. The World Bank. Worldwide Governance Indicators. Available from: http://info.worldbank.org/governance/wgi/#home [accessed 15 Jan. 2023].

3. Hale T, Angrist N, Goldszmidt R, Kira B, Petherick A, Phillips T, et al. A global panel database of pandemic policies (Oxford COVID-19 Government Response Tracker). Nature Human Behaviour. 2021;5:529-38.

4. Data on COVID-19 (coronavirus) vaccinations by Our World in Data. 2020. Available from: https://github.com/owid/covid-19-data/tree/master/public/data/vaccinations [accessed 15 Jan. 2023].

Table S5.1 Results from mobility model, assuming country fixed effects

Y: daily mobility change (%)	**Retail and recreation**			**Grocery and pharmacy**			**Transit stations**			**Workplaces**		
	Coef.	SE	*P*>z	Coef.	SE	*P*>z	Coef.	SE	*P*>z	Coef.	SE	*P*>z
Panel A: all countries												
No. of people vaccinated (per ten people)	3.59	0.25	<0.001	4.28	0.32	<0.001	2.72	0.28	<0.001	1.38	0.16	<0.001
Stringency index	-0.78	0.03	<0.001	-0.57	0.04	<0.001	-0.76	0.03	<0.001	-0.46	0.02	<0.001
No. of new cases (per 10,000 people, 1 lag)	-0.46	0.09	<0.001	-0.25	0.09	<0.001	-0.45	0.09	<0.001	-0.17	0.05	0.001
N (No. of countries)	*75,643 (107)*			*75,633 (107)*			*75,578 (107)*			*75,587 (107)*		
Panel B: high income countries												
No. of people vaccinated (per ten people)	2.73	0.25	<0.001	3.24	0.28	<0.001	2.04	0.31	<0.001	0.96	0.17	<0.001
Stringency index	-0.72	0.03	<0.001	-0.34	0.03	<0.001	-0.63	0.03	<0.001	-0.43	0.02	<0.001
No. of new cases (per 10,000 people, l lag)	-0.31	0.08	<0.001	-0.21	0.06	0.001	-0.36	0.07	<0.001	-0.10	0.04	0.017
N (No. of countries)	*35,582 (50)*			*35,576 (50)*			*35,557 (50)*			*35,579 (50)*		
Panel C: upper-middle income countries												
No. of people vaccinated (per ten people)	4.93	0.58	<0.001	5.98	0.58	<0.001	4.05	0.57	<0.001	2.08	0.35	<0.001
Stringency index	-0.69	0.05	<0.001	-0.56	0.05	<0.001	-0.74	0.05	<0.001	-0.45	0.03	<0.001
No. of new cases (per 10,000 people, l lag)	-0.65	0.20	0.003	0.15	0.20	0.465	-0.61	0.17	0.001	-0.39	0.16	0.019
N (No. of countries)	*21,074 (30)*			*21,076 (30)*			*21,040 (30)*			*21,112 (30)*		
Panel D: lower-middle income countries												
No. of people vaccinated (per ten people)	6.00	0.81	<0.001	7.90	1.32	<0.001	4.50	0.90	<0.001	2.54	0.61	<0.001
Stringency index	-0.89	0.06	<0.001	-0.90	0.07	<0.001	-0.93	0.08	<0.001	-0.50	0.06	<0.001
No. of new cases (per 10,000 people, 1 lag)	-0.21	0.43	0.636	1.24	0.54	0.031	0.52	0.54	0.350	0.26	0.36	0.473
N (No. of countries)	18,987 (27)			18,981 (27)			18,981 (27)			18,896 (27)		

Note: Fixed-effects models were applied. Other covariates not shown in the table were: population density, the share of people aged 65 or over, GDP per capita, Gini index, % female population, and indicators for continent and season. Countries were categorized into three income groups based on the 2020 per capita gross national income. Standard errors were clustered at the country level.

Table S5.2 Results from mobility model, using the 7-day moving average for mobility outcome

Y: mobility change, 7-day moving average (%)	**Retail and recreation**			**Grocery and pharmacy**			**Transit stations**			**Workplaces**		
	Coef.	SE	*P*>z	Coef.	SE	*P*>z	Coef.	SE	*P*>z	Coef.	SE	*P*>z
Panel A: all countries												
No. of people vaccinated (per ten people)	3.53	0.25	<0.001	4.28	0.32	<0.001	2.68	0.28	<0.001	1.32	0.16	<0.001
Stringency index	-0.78	0.03	<0.001	-0.56	0.04	<0.001	-0.76	0.03	<0.001	-0.46	0.02	<0.001
No. of new cases (per 10,000 people, 1 lag)	-0.44	0.09	<0.001	-0.23	0.09	0.008	-0.44	0.08	<0.001	-0.17	0.05	0.001
N (No. of countries)	*75,687 (107)*			*75,670 (107)*			*75,611 (107)*			*75,602 (107)*		
Panel B: high income countries												
No. of people vaccinated (per ten people)	2.68	0.24	<0.001	3.24	0.28	<0.001	1.99	0.31	<0.001	0.92	0.17	<0.001
Stringency index	-0.72	0.03	<0.001	-0.33	0.03	<0.001	-0.64	0.04	<0.001	-0.43	0.02	<0.001
No. of new cases (per 10,000 people, l lag)	-0.29	0.07	<0.001	-0.20	0.05	<0.001	-0.34	0.07	<0.001	-0.11	0.05	0.016
N (No. of countries)	*35,582 (50)*			*35,582 (50)*			*35,563 (50)*			*35,582 (50)*		
Panel C: upper-middle income countries												
No. of people vaccinated (per ten people)	4.84	0.58	<0.001	5.98	0.57	<0.001	3.98	0.57	<0.001	1.99	0.35	<0.001
Stringency index	-0.70	0.04	<0.001	-0.55	0.05	<0.001	-0.75	0.05	<0.001	-0.45	0.03	<0.001
No. of new cases (per 10,000 people, l lag)	-0.62	0.20	0.002	0.09	0.18	0.606	-0.59	0.17	0.001	-0.40	0.16	0.010
N (No. of countries)	*21,099 (30)*			*21,101 (30)*			*21,061 (30)*			*21,118 (30)*		
Panel D: lower-middle income countries												
No. of people vaccinated (per ten people)	5.91	0.82	<0.001	7.87	1.33	<0.001	4.44	0.91	<0.001	2.43	0.61	<0.001
Stringency index	-0.88	0.06	<0.001	-0.89	0.07	<0.001	-0.92	0.08	<0.001	-0.50	0.06	<0.001
No. of new cases (per 10,000 people, 1 lag)	-0.15	0.41	0.705	1.32	0.52	0.012	0.62	0.52	0.227	0.30	0.39	0.443
N (No. of countries)	*19,006 (27)*			*18,987 (27)*			*18,987 (27)*			*18,902 (27)*		

Note: Random-effects models were applied. The included common covariates not shown in the table were population density, the share of people aged 65 or over, GDP per capita, Gini index, % female population, and indicators for continent and season. Countries were categorized into three income groups based on the 2020 per capita gross national income. Standard errors were clustered at the country level.

Table S5.3 Results from mobility model: using the lagged vaccine coverage as key predictor

Y: daily mobility change (%)	**Retail and recreation**			**Grocery and pharmacy**			**Transit stations**			**Workplaces**		
	Coef.	SE	*P*>z	Coef.	SE	*P*>z	Coef.	SE	*P*>z	Coef.	SE	*P*>z
Panel A: all countries												
No. of people vaccinated (per ten people, 7 lags)	3.55	0.26	<0.001	4.17	0.33	<0.001	2.68	0.28	<0.001	1.38	0.17	<0.001
Stringency index	-0.80	0.03	<0.001	-0.61	0.04	<0.001	-0.78	0.04	<0.001	-0.46	0.02	<0.001
No. of new cases (per 10,000 people, 1 lag)	-0.47	0.09	<0.001	-0.25	0.09	0.006	-0.45	0.09	<0.001	-0.16	0.05	0.001
N (No. of countries)	*75,006 (107)*			*74,996 (107)*			*74,941 (107)*			*74,950 (107)*		
Panel B: high income countries												
No. of people vaccinated (per ten people, 7 lags)	2.72	0.25	<0.001	3.21	0.28	<0.001	2.04	0.32	<0.001	0.96	0.17	<0.001
Stringency index	-0.73	0.04	<0.001	-0.35	0.04	<0.001	-0.63	0.04	<0.001	-0.42	0.03	<0.001
No. of new cases (per 10,000 people, l lag)	-0.32	0.08	<0.001	-0.21	0.06	<0.001	-0.36	0.07	<0.001	-0.10	0.04	0.016
N (No. of countries)	*35,282 (50)*			*35,276 (50)*			*35,257 (50)*			*35,279 (50)*		
Panel C: upper-middle income countries												
No. of people vaccinated (per ten people, 7 lags)	4.91	0.58	<0.001	5.86	0.58	<0.001	4.04	0.55	<0.001	2.13	0.35	<0.001
Stringency index	-0.72	0.05	<0.001	-0.60	0.05	<0.001	-0.76	0.06	<0.001	-0.44	0.03	<0.001
No. of new cases (per 10,000 people, l lag)	-0.65	0.20	0.001	0.14	0.19	0.473	-0.61	0.17	<0.001	-0.38	0.16	0.015
N (No. of countries)	*20,895 (30)*			*20,897 (30)*			*20,861 (30)*			*20,933 (30)*		
Panel D: lower-middle income countries												
No. of people vaccinated (per ten people, 7 lags)	6.03	0.81	<0.001	7.86	1.31	<0.001	4.49	0.90	<0.001	2.61	0.63	<0.001
Stringency index	-0.91	0.06	<0.001	-0.95	0.07	<0.001	-0.96	0.08	<0.001	-0.51	0.07	<0.001
No. of new cases (per 10,000 people, 1 lag)	-0.24	0.41	0.554	1.19	0.52	0.022	0.48	0.52	0.353	0.24	0.35	0.483
N (No. of countries)	*18,829 (27)*			*18,823 (27)*			*18,823 (27)*			*18,738 (27)*		

Note: Random-effects models were applied. The included common covariates not shown in the table were population density, the share of people aged 65 or over, GDP per capita, Gini index, % female population, and indicators for continent and season. Countries were categorized into three income groups based on the 2020 per capita gross national income. Standard errors were clustered at the country level.

Table S5.4 Results from mobility model: using instrumental variables for vaccine coverage

Y: daily mobility change (%)	Retail and recreation			Grocery and pharmacy			Transit stations			Workplaces		
	Coef.	SE	*P*>z	Coef.	SE	*P*>z	Coef.	SE	*P*>z	Coef.	SE	*P*>z
Panel A: all countries												
No. of people vaccinated (per ten people)	4.35	0.37	<.001	5.55	0.51	<.001	3.56	0.40	<.001	1.46	0.21	<.001
Stringency index	-0.75	0.03	<.001	-0.53	0.04	<.001	-0.74	0.03	<.001	-0.46	0.02	<.001
No. of new cases (per 10,000 people, 1 lag)	-0.57	0.12	<.001	-0.38	0.13	0.002	-0.56	0.11	<.001	-0.17	0.06	0.003
N (No. of countries)	*74,836 (107)*			*74,826 (107)*			*74,771 (107)*			*74,793 (107)*		
Panel B: high income countries												
No. of people vaccinated (per ten people)	2.92	0.27	<.001	3.67	0.33	<.001	2.40	0.38	<.001	1.02	0.19	<.001
Stringency index	-0.72	0.03	<.001	-0.32	0.03	<.001	-0.62	0.03	<.001	-0.43	0.02	<.001
No. of new cases (per 10,000 people, l lag)	-0.34	0.09	<.001	-0.25	0.07	<.001	-0.40	0.08	<.001	-0.11	0.04	0.011
N (No. of countries)	*35,262 (50)*			*35,256 (50)*			*35,237 (50)*			*35,259 (50)*		
Panel C: upper-middle income countries												
No. of people vaccinated (per ten people)	6.19	1.06	<.001	8.00	1.53	<.001	5.41	1.07	<.001	2.30	0.50	<.001
Stringency index	-0.65	0.05	<.001	-0.48	0.07	<.001	-0.69	0.07	<.001	-0.44	0.03	<.001
No. of new cases (per 10,000 people, l lag)	-0.94	0.28	0.001	-0.13	0.36	0.721	-0.91	0.25	<.001	-0.50	0.18	0.005
N (No. of countries)	*20,880 (30)*			*20,882 (30)*			*20,846 (30)*			*20,918 (30)*		
Panel D: lower-middle income countries												
No. of people vaccinated (per ten people)	8.38	1.38	<.001	10.78	1.96	<.001	6.26	1.26	<.001	2.72	0.87	0.002
Stringency index	-0.86	0.06	<.001	-0.86	0.07	<.001	-0.91	0.08	<.001	-0.50	0.06	<.001
No. of new cases (per 10,000 people, 1 lag)	-0.98	0.69	0.153	0.31	0.89	0.724	-0.05	0.77	0.948	0.21	0.45	0.636
N (No. of countries)	*18,694 (27)*			*18,688 (27)*			*18,688 (27)*			*18,616 (27)*		

Note: Random-effects models were applied. Vaccine coverage was instrumented by the government effectiveness score and an ordinal variable for COVID-19 vaccine availability. The included common covariates not shown in the table were population density, the share of people aged 65 or over, GDP per capita, Gini index, % female population, and indicators for continent and season. Countries were categorized into three income groups based on the 2020 per capita gross national income. Standard errors were clustered at the country level.

Table S5.5 Results from mobility model: adding the number of boosters and interaction with vaccine coverage

	**Retail and recreation**			**Grocery and pharmacy**			**Transit stations**			**Workplaces**		
Y: daily mobility change (%)	Coef.	SE	*P*>z	Coef.	SE	*P*>z	Coef.	SE	*P*>z	Coef.	SE	*P*>z
Sample: all countries												
No. of people vaccinated (per ten people)	3.90	0.30	<0.001	4.75	0.37	<0.001	3.05	0.35	<0.001	1.54	0.19	<0.001
No. of boosters (per ten fully vaccinated people)	0.96	2.23	0.666	5.06	2.31	0.029	-0.35	2.99	0.906	0.16	1.58	0.921
No. of people vaccinated × No. of boosters	-0.38	0.27	0.162	-1.06	0.31	0.001	-0.27	0.39	0.495	-0.13	0.21	0.523
Stringency index	-0.79	0.03	<0.001	-0.58	0.04	<0.001	-0.77	0.03	<0.001	-0.46	0.02	<0.001
No. of new cases (per 10,000 people, 1 lag)	-0.32	0.08	<0.001	-0.03	0.09	0.735	-0.25	0.08	0.002	-0.11	0.05	0.026
	dy/dx	SE	*P*>z	dy/dx	SE	*P*>z	dy/dx	SE	*P*>z	dy/dx	SE	*P*>z
Average marginal effects			[95% CI]			[95% CI]			[95% CI]			[95% CI]
No. of people vaccinated (per ten people)	3.80	0.31	<0.001	4.47	0.38	<0.001	2.98	0.36	<0.001	1.50	0.20	<0.001
			[3.19, 4.41]			[3.73, 5.21]			[2.27, 3.70]			[1.12, 1.89]
No. of boosters (per ten fully vaccinated people)	0.27	1.75	0.879	3.13	1.78	0.078	-0.84	2.29	0.715	-0.08	1.21	0.944
			[-3.16, 3.69]			[-0.36, 6.61]			[-5.33, 3.66]			[-2.46, 2.29]
N (No. of countries)			*72,145 (102)*			*72,135 (102)*			*72,080 (102)*			*72,089 (102)*

Note: Random-effects models were applied. The included common covariates not shown in the table were population density, the share of people aged 65 or over, GDP per capita, Gini index, % female population, and indicators for continent and season. Countries were categorized into three income groups based on the 2020 per capita gross national income. Standard errors were clustered at the country level. The average marginal effects were obtained by taking the derivatives of mobility with respect to corresponding predictors (dy/dx). The Delta-method was used to calculate the standard errors.

Appendix S6 Robustness check for case growth model

For robustness check of case growth model, we replaced the case growth with the reproduction rate, obtained from Our World in Data (OWID) [1]. The results are presented in Table S6.1. The difference in vaccine coverage coefficient between the base and full models is indicated in italics. For the full model with the mobility index, the coefficient difference was −0.004 (P = 0.006). Similarly, after adjustment for mobility index and mobility in retail stores, grocery stores, and transit stations, the difference in coefficient of vaccine coverage was −0.007 (P < 0.001), −0.002 (P = 0.024), and −0.005 (P = 0.003) percentage points, respectively. In other words, mobility in retail stores, grocery stores, and transit stations attenuated vaccine effectiveness in curbing case growth by 30% (0.007/0.023), 11% (0.002/0.018), and 25% (0.005/0.02), respectively. Only workplace mobility exhibited no mediating role in the vaccine–infection relationship. These results appeared to be consistent with those from the primary models.

We further investigated whether the conclusions about vaccine-infection relationship still held, after adjusting the number different COVID-19 vaccines in the case growth model. The rationale was that, independent of the vaccine coverage, which measures the breadth (population covered), the depth (average dose number) of vaccination may also affect the case growth. To investigate the topic, we collected information on the dates of Emergency Use Listing for different vaccines approved by the World Health Organization (WHO), US Food and Drug Administration (FDA), and the European Medicines Agency (EMA). The related information is presented in Table S6.2. However, we did not find the number of individual vaccines for all 107 countries analysed in this study. The best available data source appeared to be "Covid-19 vaccine doses administered by manufacturer", provided by Our World in Data (OWID)[2]. The dataset tracks the cumulative number of doses administered, broken down by 10 vaccine manufacturers, for 43 countries (including 27 European Union countries) per day, from as early as 4 December 2020 to the most recent date. The ten vaccines are: Pfizer/BioNTech (BNT), Moderna, Oxford/AstraZeneca (AZ), Johnson & Johnson, Sinopharm/Beijing, Sputnik V, Sinovac, Novavax, Covaxin, and CanSino. For empirical analysis, we excluded three countries (Cyprus, Iceland and Liechtenstein) that were not in the study sample, and two countries (Nepal, South Africa) for which their vaccine data by manufacturer were not available during the study period. The final sample was composed of 38 countries.

We reported the cumulative number of doses of different vaccines as of 6 February 2022 (the last day of the study period) for 38 countries in Table S6.3. In this analysis, the first day of the study period was the earliest date when the vaccine type data were available, which was approximately the first day of vaccine rollout and varied across countries. Three vaccines (Novavax, Covaxin, and CanSino) were not reported due to their low numbers. As the last row shows, BNT, Moderna, and AZ were top three vaccines, accounting for 92.1% of vaccine doses administered.

Because other vaccines were either had a relatively small number of doses, or approved at a later date, we focused on the three major vaccines. The interest was the association between daily case growth rate and the number of three different vaccines. Therefore, three corresponding variables were included in case growth model: the number of BNT, Moderna, and AZ vaccines administrated per 10 vaccinated people, respectively. For example, the number of BNT vaccines was calculated as:

$$\frac{\text{Cumulative number of BNT vaccine doses administrated in country } i \text{ on day } t}{\text{Cumulative number of people received at least one dose of COVID} - 19 \text{ vaccines in country } i \text{ on day } t} \times 10$$

In line with the calculation of effective vaccine coverage in the case growth model, we used the effective vaccine number, i.e., the number from 7 days and 6 months ago for all three vaccine types. The results are presented in Table S6.4. The full models show that, the daily case growth rate decreased by 0.19-0.21 (P < 0.001) and 0.26-0.29 percentage points (P < 0.005) when the effective number of BNT and AZ vaccines per 10 vaccinated people increased by one, respectively.

However, the number of Moderna vaccines was not associated with the case growth after adjusting for the vaccine coverage, human mobility, the number of BNT and AZ vaccines, stringency index and a series of control variables. The results should be interpreted with caution because these were aggregated data. Due to data limitation, we did not control for other variables that could affect the effectiveness of different vaccines, such as the duration between vaccination, the timing of Emergency Use Listing, and the sequence of different vaccines received by individuals.

We examined whether the conclusion that mobility weakened the negative relationship between vaccination and case growth remained robust when the new predictors were included. As Table S6.4 shows, after adjustment for mobility in retail stores and transit stations, the difference in coefficient of vaccine coverage between the base and full models was −0.15 (P = 0.015) and −0.103 (P = 0.026) percentage points, respectively. In other words, mobility in retail stores and transit stations attenuated vaccine effectiveness in curbing case growth by 32.6% (0.15/0.46) and 24.4% (0.1/0.41), respectively. Mobility in grocery stores and workplaces exhibited no mediating role in the vaccine–infection relationship. These results suggested that the findings were similar to those from the primary models, except that mobility in grocery stores did not appear to attenuate vaccine effectiveness in 38 countries studied, where 33 were high-income.

References:

1. Coronavirus Pandemic. 2020. Available from: https://ourworldindata.org/coronavirus [accessed 19 Sep. 2022].

2. COVID-19 vaccine doses administered by manufacturer, European Union. 2023. Available from: https://ourworldindata.org/grapher/covid-vaccine-doses-by-manufacturer [accessed 18 Jan. 2023].

Table S6.1 Results from case growth model, using the reproduction rate as outcome

Y: Reproduction rate	Coefficient	[95% CI]	*P*>z
**Base model**			
Effective vaccine coverage	-0.016	[-0.029 , -0.002]	0.024
Stringency index (14 lags)	-0.004	[-0.006 , -0.003]	<0.001
**Full model, with mobility Index**			
Effective vaccine coverage	-0.020	[-0.034 , -0.006]	0.005
Mobility index (14 lags)	0.002	[0.001 , 0.003]	<0.001
Stringency index (14 lags)	-0.003	[-0.005 , -0.002]	<0.001
Diff. in coef. of vaccine coverage	-0.004	[-0.007 , -0.001]	0.006
**Full model, with retail**			
Effective vaccine coverage	-0.023	[-0.036 , -0.009]	0.001
Mobility: retail & recreation (14 lags)	0.002	[0.002 , 0.003]	<0.001
Stringency index (14 lags)	-0.003	[-0.005 , -0.001]	0.001
Diff. in coef. of vaccine coverage	-0.007	[-0.010 , -0.003]	<0.001
**Full model, with grocery**			
Effective vaccine coverage	-0.018	[-0.031 , -0.004]	0.011
Mobility: grocery & pharmacy (14 lags)	0.001	[0.000 , 0.002]	0.004
Stringency index (14 lags)	-0.004	[-0.006 , -0.002]	<0.001
Diff. in coef. of vaccine coverage	-0.002	[-0.004 , 0.000]	0.024
**Full model, with transit stations**			
Effective vaccine coverage	-0.020	[-0.034 , -0.006]	0.005
Mobility: transit stations (14 lags)	0.002	[0.001 , 0.003]	0.001
Stringency index (14 lags)	-0.003	[-0.005 , -0.002]	<0.001
Diff. in coef. of vaccine coverage	-0.005	[-0.008 , -0.002]	0.003
**Full model, with workplaces**			
Effective vaccine coverage	-0.016	[-0.030 , -0.002]	0.021
Mobility: workplaces (14 lags)	0.000	[0.000 , 0.001]	0.273
Stringency index (14 lags)	-0.004	[-0.006 , -0.003]	<0.001
Diff. in coef. of vaccine coverage	0.000	[-0.001 , 0.000]	0.337

Note: The full models included mobility variables while the base model did not. Effective vaccine coverage was defined as the cumulative number of people who received at least one dose of COVID-19 vaccines per ten people from 7 days and 6 months ago. The sample was a longitudinal dataset comprising data from 107 countries with 39,450 country-days. Only the postvaccination period was used for analysis. The included common covariates not shown in the table were face-covering policies, indicators for season, Delta period, and Omicron period. Fixed-effects models were applied; standard errors were clustered at the country level.

Table S6.2 COVID-19 vaccines granted Emergency Use Listing by WHO, FDA, and EMA

Manufacturer	Date of Emergency Use Listing		
	World Health Organization (WHO)	US Food and Drug Administration (FDA)	European Medicines Agency (EMA)
Pfizer/BioNTech	31 Dec. 2020	11 Dec. 2020	21 Dec. 2020
Oxford/AstraZeneca	15 Feb. 2021	N/A	29 Jan. 2021
Moderna	30 Apr. 2021	18 Dec. 2020	6 Jan. 2021
Johnson & Johnson	12 Mar. 2021	27 Feb 2021	11 Jan. 2021
Sinopharm/Beijing	7 May. 2021	N/A	N/A
Sinovac	1 Jun. 2021	N/A	N/A
Covaxin	3 Nov. 2021	N/A	N/A
Novavax	17 Dec. 2021	13 Jul. 2022	20 Dec. 2021
CanSino	19 May. 2022	N/A	N/A
Sputnik V	Under rolling review	N/A	Under rolling review

Note: Data sources for Emergency Use Listing (accessed 13 Jan. 2023) are as follows:
WHO: https://covid19.trackvaccines.org/agency/who/
FDA: https://www.fda.gov/emergency-preparedness-and-response/coronavirus-disease-2019-covid-19/covid-19-vaccines
EMA: https://www.ema.europa.eu/en/human-regulatory/overview/public-health-threats/coronavirus-disease-covid-19/treatments-vaccines/vaccines-covid-19/covid-19-vaccines-authorised

Table S6.3 Cumulative vaccine doses in 38 countries as of 6 February 2022, by manufacturer (unit: million)

Country	BNT	Moderna	AZ	Sinovac	Sinopharm	J&J	Sputnik V
Argentina	11,407,986	5,396,915	25,685,475	0	27,519,764	0	19,917,442
Austria	14,408,585	1,583,345	1,592,840	0	0	366,989	0
Belgium	16,958,245	4,169,744	2,848,972	0	0	427,188	0
Bulgaria	2,748,000	478,158	478,514	0	0	499,873	0
Canada	53,275,635	22,161,502	2,809,644	0	0	16,921	0
Chile	7,798,839	0	548,545	25,178,377	0	0	0
Croatia	3,859,485	502,770	568,444	0	0	198,127	0
Czechia	14,332,997	1,552,067	886,768	309	57	410,994	0
Denmark	10,193,073	1,674,290	155,938	0	0	46,025	0
Ecuador	8,552,679	0	5,009,163	15,812,935	0	0	0
Estonia	1,472,792	228,692	238,906	0	0	77,289	0
Finland	9,018,975	1,852,227	553,895	0	0	0	0
France	106,680,326	22,915,237	7,862,609	0	0	1,088,142	0
Germany	124,804,502	29,259,042	12,784,827	0	0	3,728,545	0
Hong Kong	6,979,622	0	0	4,318,547	0	0	0
Hungary	106,680,326	22,915,237	7,862,609	0	0	1,088,142	0
Ireland	9,264,375	1,045,546	1,252,732	0	2,286,612	330,787	1,807,392
Italy	7,870,659	1,185,023	1,215,315	0	0	241,277	0
Japan	177,765,742	34,187,148	116,772	0	0	0	0
Latvia	85,053,370	31,779,501	12,169,368	0	0	1,507,429	0
Lithuania	1,568,134	688,632	262,028	9	27	291,117	0
Luxembourg	3,260,506	326,230	536,489	0	0	293,416	0
Malta	688,718	290,706	105,059	0	0	41,390	0
Netherlands	693,504	229,625	227,769	0	0	32,192	0
Norway	21,438,556	6,257,686	2,473,242	0	0	752,609	0
Peru	34,505,234	0	4,636,066	0	19,907,081	0	0
Poland	8,732,787	2,242,953	147,920	0	0	7,281	0
Portugal	37,497,535	3,589,959	5,292,114	0	0	2,643,304	0
Romania	14,566,312	3,335,175	2,270,464	7,429	3,907	1,136,552	0
Slovakia	12,647,268	995,412	849,551	0	0	2,024,701	0
Slovenia	5,026,530	670,669	844,164	0	0	183,252	37,984
South Korea	51,987,903	13,439,677	20,323,913	0	0	1,516,502	0
Spain	2,185,165	232,925	323,098	0	0	135,792	0
Sweden	62,991,908	22,432,291	9,794,903	0	0	1,981,322	0
Switzerland	5,675,749	9,661,946	0	0	0	58,854	0
Ukraine	14,045,627	2,980,084	4,029,295	9,642,922	0	20,680	0
United States	319,082,480	205,409,451	0	0	0	18,246,402	0
Uruguay	2,275,663	0	91,128	3,248,118	0	0	0
Total	1,377,995,792	455,669,865	136,848,539	58,208,646	49,717,448	39,393,094	21,762,818

Source: COVID-19 vaccine doses administered by manufacturer, European Union (2023). Available from: https://ourworldindata.org/grapher/covid-vaccine-doses-by-manufacturer. [cited 18 Jan. 2023]

Table S6.4 Results from case growth model: adding number of different COVID-19 vaccines

Y: Case growth (%)	Coefficient	[95% CI]	*P*>z
**Base model**			
Effective vaccine coverage	-0.310	[-0.505, -0.115]	0.002
No. of BNT vaccines (per 10 vacc. people)	-0.214	[-0.316, -0.112]	<0.001
No. of Moderna vaccines (per 10 vacc. people)	0.074	[-0.224, 0.372]	0.627
No. of AZ vaccines (per 10 vacc. people)	-0.259	[-0.432, -0.085]	0.003
Stringency index (14 lags)	-0.099	[-0.130, -0.067]	<0.001
**Full model, with mobility Index**			
Effective vaccine coverage	-0.397	[-0.567, -0.227]	<0.001
Mobility index (14 lags)	0.040	[0.011, 0.069]	0.007
No. of BNT vaccines (per 10 vacc. people)	-0.209	[-0.312, -0.107]	<0.001
No. of Moderna vaccines (per 10 vacc. people)	0.072	[-0.218, 0.362]	0.626
No. of AZ vaccines (per 10 vacc. people)	-0.279	[-0.432, -0.126]	<0.001
Stringency index (14 lags)	-0.081	[-0.113, -0.048]	<0.001
Diff. in coef. of vaccine coverage	-0.087	[-0.169, -0.005]	0.038
**Full model, with retail**			
Effective vaccine coverage	-0.460	[-0.638, -0.281]	<0.001
Mobility: retail & recreation (14 lags)	0.055	[0.023, 0.086]	0.001
No. of BNT vaccines (per 10 vacc. people)	-0.193	[-0.293, -0.093]	<0.001
No. of Moderna vaccines (per 10 vacc. people)	0.067	[-0.242, 0.375]	0.672
No. of AZ vaccines (per 10 vacc. people)	-0.288	[-0.439, -0.137]	<0.001
Stringency index (14 lags)	-0.064	[-0.097, -0.030]	<0.001
Diff. in coef. of vaccine coverage	-0.150	[-0.271, -0.030]	0.015
**Full model, with grocery**			
Effective vaccine coverage	-0.350	[-0.525, -0.175]	<0.001
Mobility: grocery & pharmacy (14 lags)	0.019	[0.000, 0.039]	0.052
No. of BNT vaccines (per 10 vacc. people)	-0.213	[-0.314, -0.112]	<0.001
No. of Moderna vaccines (per 10 vacc. people)	0.075	[-0.217, 0.366]	0.617
No. of AZ vaccines (per 10 vacc. people)	-0.271	[-0.433, -0.110]	0.001
Stringency index (14 lags)	-0.093	[-0.124, -0.062]	<0.001
Diff. in coef. of vaccine coverage	-0.040	[-0.091, 0.011]	0.122
**Full model, with transit stations**			
Effective vaccine coverage	-0.413	[-0.585, -0.241]	<0.001
Mobility: transit stations (14 lags)	0.045	[0.012, 0.078]	0.008
No. of BNT vaccines (per 10 vacc. people)	-0.200	[-0.304, -0.096]	<0.001
No. of Moderna vaccines (per 10 vacc. people)	0.063	[-0.232, 0.358]	0.676
No. of AZ vaccines (per 10 vacc. people)	-0.275	[-0.428, -0.122]	<0.001
Stringency index (14 lags)	-0.073	[-0.108, -0.038]	<0.001
Diff. in coef. of vaccine coverage	-0.103	[-0.195, -0.012]	0.026
**Full model, with workplaces**			
Effective vaccine coverage	-0.312	[-0.502, -0.122]	0.001
Mobility: workplaces (14 lags)	0.001	[-0.015, 0.018]	0.859
No. of BNT vaccines (per 10 vacc. people)	-0.214	[-0.314, -0.114]	<0.001
No. of Moderna vaccines (per 10 vacc. people)	0.074	[-0.222, 0.371]	0.624
No. of AZ vaccines (per 10 vacc. people)	-0.259	[-0.429, -0.090]	0.003
Stringency index (14 lags)	-0.098	[-0.130, -0.066]	<0.001
Diff. in coef. of vaccine coverage	-0.002	[-0.028, 0.023]	0.861

Note: The full models included mobility variables while the base model did not. Effective vaccine coverage was defined as the cumulative number of people who received at least one dose of COVID-19 vaccines per ten people from 7 days and 6 months ago. Number of BNT/Moderna/AZ vaccines was defined as the number of BNT/ Moderna/AZ vaccine doses administered per 10 vaccinated people. The sample was a longitudinal dataset comprising data from 38 countries (including 27 European Union countries) and had 7,426 country-days. Only the postvaccination period was used for analysis. The included common covariates not shown in the table were face-covering policies, indicators for season, Delta period, and Omicron period. Fixed-effects models were applied; standard errors were clustered at the country level. Abbreviations: BNT=Pfizer/BioNTech; AZ=Oxford/AstraZeneca; vacc.=vaccinated.

Appendix S7 Mobility model: adding interactions between vaccine coverage rate and stage of vaccination

Table S7 Results from mobility model: adding interactions between vaccine coverage rate and stage of vaccination

Y: daily mobility change (%)	**Retail and recreation**			**Grocery and pharmacy**			**Transit stations**			**Workplaces**		
	Coef.	SE	*P*>z	Coef.	SE	*P*>z	Coef.	SE	*P*>z	Coef.	SE	*P*>z
Panel A: all countries												
No. of people vaccinated (per ten people)												
× Stage 1	10.83	1.53	<0.001	19.17	2.08	<0.001	10.70	1.39	<0.001	3.51	0.87	<0.001
× Stage 2	5.93	0.56	<0.001	8.92	0.69	<0.001	5.32	0.59	<0.001	2.63	0.38	<0.001
× Stage 3	3.98	0.30	<0.001	5.32	0.34	<0.001	3.51	0.46	<0.001	1.37	0.19	<0.001
× Stage 4	3.57	0.31	<0.001	4.01	0.38	<0.001	2.48	0.31	<0.001	1.41	0.21	<0.001
× Stage 5	2.55	0.27	<0.001	2.73	0.34	<0.001	1.87	0.32	<0.001	0.83	0.22	<0.001
Stringency index	-0.77	0.03	<0.001	-0.56	0.04	<0.001	-0.75	0.03	<0.001	-0.46	0.02	<0.001
No. of new cases (per 10,000 people, 1 lag)	-0.45	0.09	<0.001	-0.21	0.08	0.009	-0.43	0.08	<0.001	-0.16	0.05	0.001
N (No. of countries)	*75,643 (107)*			*75,633 (107)*			*75,578 (107)*			*75,587 (107)*		
Panel B: high income countries												
No. of people vaccinated (per ten people)												
× Stage 1	5.75	1.09	<0.001	10.74	1.21	<0.001	6.26	1.22	<0.001	5.36	0.97	<0.001
× Stage 2	4.60	0.52	<0.001	6.27	0.55	<0.001	4.22	0.59	<0.001	2.51	0.39	<0.001
× Stage 3	3.28	0.32	<0.001	4.45	0.38	<0.001	3.03	0.63	<0.001	1.10	0.18	<0.001
× Stage 4	2.66	0.31	<0.001	2.95	0.31	<0.001	1.71	0.32	<0.001	0.93	0.23	<0.001
× Stage 5	2.21	0.30	<0.001	2.69	0.40	<0.001	1.85	0.38	<0.001	0.75	0.25	0.003
Stringency index	-0.73	0.04	<0.001	-0.36	0.04	<0.001	-0.64	0.04	<0.001	-0.44	0.03	<0.001
No. of new cases (per 10,000 people, l lag)	-0.29	0.08	<0.001	-0.17	0.05	0.001	-0.33	0.07	<0.001	-0.10	0.04	0.021
N (No. of countries)	*35,582 (50)*			*35,576 (50)*			*35,557 (50)*			*35,579 (50)*		
Panel C: upper-middle income countries												
No. of people vaccinated (per ten people)												
× Stage 1	14.00	3.09	<0.001	22.07	4.44	<0.001	14.19	2.75	<0.001	2.75	1.62	0.090
× Stage 2	5.94	1.02	<0.001	9.29	1.07	<0.001	5.22	1.07	<0.001	1.93	0.71	0.006
× Stage 3	4.04	0.65	<0.001	5.55	0.61	<0.001	3.68	0.80	<0.001	1.44	0.38	<0.001
× Stage 4	5.22	0.65	<0.001	5.86	0.62	<0.001	4.14	0.56	<0.001	2.40	0.43	<0.001
× Stage 5	4.31	0.83	<0.001	4.27	0.83	<0.001	3.23	0.60	<0.001	1.35	0.79	0.088

Stringency index	-0.68	0.04	<0.001	-0.51	0.05	<0.001	-0.72	0.05	<0.001	-0.45	0.03	<0.001
No. of new cases (per 10,000 people, l lag)	-0.74	0.18	<0.001	-0.09	0.17	0.621	-0.72	0.17	<0.001	-0.34	0.14	0.017
N (No. of countries)	*21,074 (30)*			*21,076 (30)*			*21,040 (30)*			*21,112 (30)*		
Panel D: lower-middle income countries												
No. of people vaccinated (per ten people)												
× Stage 1	15.15	3.43	<0.001	23.60	4.27	<0.001	11.87	2.99	<0.001	2.30	2.17	0.291
× Stage 2	9.22	1.48	<0.001	12.56	1.88	<0.001	7.60	1.71	<0.001	4.24	0.99	<0.001
× Stage 3	5.80	0.72	<0.001	7.86	0.95	<0.001	4.58	0.81	<0.001	2.30	0.68	0.001
× Stage 4	5.57	0.71	<0.001	7.45	1.25	<0.001	4.17	0.89	<0.001	2.60	0.59	<0.001
× Stage 5	3.62	0.25	<0.001	3.03	0.76	<0.001	1.34	0.37	<0.001	0.56	0.14	<0.001
Stringency index	-0.85	0.06	<0.001	-0.84	0.07	<0.001	-0.90	0.08	<0.001	-0.50	0.06	<0.001
No. of new cases (per 10,000 people, 1 lag)	-0.17	0.46	0.717	1.23	0.53	0.021	0.53	0.54	0.324	0.25	0.31	0.408
N (No. of countries)	*18,987 (27)*			*18,981 (27)*			*18,981 (27)*			*18,896 (27)*		

Note: Vaccination stage was determined on the basis of the proportion of the population receiving at least one dose of COVID-19 vaccines: above 0 and ≤20% (Stage 1), 20%–40% (Stage 2), 40%– 60% (Stage 3), 60%–80% (Stage 4), and ≥80% (Stage 5); the reference stage is the prevaccination period. This table was used to produce Figure 2 in the main text. Random-effects models were applied. The included common covariates not shown in the table were population density, the share of people aged 65 or over, GDP per capita, Gini index, % female population, and indicators for continent and season. Standard errors were clustered at the country level.

Appendix S8 Applying false-positive corrections to mobility and case growth models

In order to reduce the likelihood of false rejections in our models, we employed Anderson's method and calculated the False Discovery Rate (FDR) q-values [1]. It is the ratio of the number of false positive results to the number of total positive test results. Anderson's method takes *P*-values as inputs as in a Bonferroni adjustment, and has greater power than other alternatives [2].

The adjusted *P*-values, or *Q*-values, are presented in Table S8.1 for key covariates in the mobility model, and in Table S8.2 for the case growth model. The results showed that most of the adjusted *P*-values were larger than the original *P*-values. However, they were still very small, with all of the adjusted *P*-values for vaccine coverage smaller than 0.005. Therefore, the conclusions drawn from the original models remained robust. It is noteworthy that some adjusted *P*-values were actually less than the original values (9 out of 48 in Table S8.1 and 1 out of 17 in Table S8.2). The reason for this is that if there are many true rejections, one can tolerate several false rejections and still keep the false discovery rate low [1, 2].

Reference:

1. Anderson ML. Multiple inference and gender differences in the effects of early intervention: a reevaluation of the Abecedarian, Perry Preschool, and Early Training Projects. Journal of the American Statistical Association. 2008;103:1481-95. DOI: 10.1198/016214508000000841
2. McKenzie D. An overview of multiple hypothesis testing commands in Stata. World Bank blogs. 1 June. 2020. Available from https://blogs.worldbank.org/impactevaluations/overview-multiple-hypothesis-testing-commands-stata. [accessed 23 Jan. 2023]

Table S8.1 Results of applying false-positive correction to mobility model presented in Table 3

Y: daily mobility change (%)	Retail and recreation			Grocery and pharmacy			Transit stations			Workplaces		
	Coef.	*P*>z	Adj. *P*	Coef.	*P*>z	Adj. *P*	Coef.	*P*>z	Adj. *P*	Coef.	*P*>z	Adj. *P*
Panel A: all countries												
No. of people vaccinated (per ten people)	3.59	0.000000000	0.001	4.28	0.000000000	0.001	2.72	0.000000000	0.001	1.38	0.000000000	0.001
Stringency index	-0.78	0.000000000	0.001	-0.57	0.000000000	0.001	-0.76	0.000000000	0.001	-0.46	0.000000000	0.001
No. of new cases (per 10,000 people, 1 lag)	-0.46	0.637928200	0.057	-0.24	0.006485000	0.002	-0.45	0.000000109	0.001	-0.17	0.000764000	0.001
N (No. of countries)		*75,643 (107)*			*75,633 (107)*			*75,578 (107)*			*75,587 (107)*	
Panel B: high income (HI) countries												
No. of people vaccinated (per ten people)	2.73	0.000000000	0.001	3.24	0.000000000	0.001	2.03	0.000000000	0.001	0.96	0.000000000	0.001
Stringency index	-0.72	0.000000000	0.001	-0.33	0.000000000	0.001	-0.63	0.000000000	0.001	-0.43	0.000000000	0.001
No. of new cases (per 10,000 people, l lag)	-0.31	0.000093951	0.001	-0.21	0.000239209	0.001	-0.36	0.000000362	0.001	-0.1	0.012870721	0.002
N (No. of countries)		*35,582 (50)*			*35,576 (50)*			*35,557 (50)*			*35,579 (50)*	
Panel C: upper-middle income countries												
No. of people vaccinated (per ten people)	4.93	0.000000000	0.001	5.98	0.000000000	0.001	4.05	0.000000000	0.001	2.07	0.000000000	0.001
Stringency index	-0.69	0.000000000	0.001	-0.56	0.000000000	0.001	-0.74	0.000000000	0.001	-0.45	0.000000000	0.001
No. of new cases (per 10,000 people, l lag)	-0.65	0.001105221	0.001	0.14	0.458328109	0.04	-0.61	0.000340073	0.001	-0.39	0.012747099	0.003
N (No. of countries)		*21,074 (30)*			*21,076 (30)*			*21,040 (30)*			*21,112 (30)*	
Panel D: lower-middle income countries												
No. of people vaccinated (per ten people)	6.00	0.000000000	0.001	7.90	0.000000637	0.001	4.50	0.000000637	0.001	2.54	0.000000000	0.001
Stringency index	-0.89	0.000000000	0.001	-0.9	0.000000000	0.001	-0.93	0.000000000	0.001	-0.5	0.000000000	0.001
No. of new cases (per 10,000 people, 1 lag)	-0.20	0.637928219	0.19	1.24	0.021073331	0.01	0.52	0.334360972	0.112	0.27	0.454832788	0.142
N (No. of countries)		*18,987 (27)*			*18,981 (27)*			*18,981 (27)*			*18,896 (27)*	

Note: The adjusted *P*-values are the False Discovery Rate (FDR) *Q*-values computed using Anderson's code, available at: https://are.berkeley.edu/~mlanderson/downloads/fdr_sharpened_qvalues.do.zip.

Table S8.2 Results of applying false-positive correction to case growth model presented in Figure 3

**Base model**	Coef.	[95% Conf. Int.]		*P*>z	Adj. *P*-value
Effective vaccine coverage	-0.28	-0.43	-0.13	0.00039359875	0.001
Stringency index (14 lags)	-0.10	-0.12	-0.07	0.00000000001	0.001
**Full model, with mobility Index**					
Effective vaccine coverage	-0.38	-0.55	-0.21	0.00002106045	0.001
Mobility index (14 lags)	0.05	0.03	0.07	0.00000343197	0.001
Stringency index (14 lags)	-0.08	-0.10	-0.05	0.00000000372	0.001
**Full model, with retail**					
Effective vaccine coverage	-0.42	-0.59	-0.25	0.00000400288	0.001
Mobility: retail & recreation (14 lags)	0.05	0.04	0.07	0.00000000520	0.001
Stringency index (14 lags)	-0.07	-0.09	-0.04	0.00000014390	0.001
**Full model, with grocery**					
Effective vaccine coverage	-0.33	-0.49	-0.18	0.00006978839	0.001
Mobility: grocery & pharmacy (14 lags)	0.03	0.01	0.04	0.00004950538	0.001
Stringency index (14 lags)	-0.09	-0.11	-0.06	0.00000000006	0.001
**Full model, with transit stations**					
Effective vaccine coverage	-0.38	-0.55	-0.22	0.00001958263	0.001
Mobility: transit stations (14 lags)	0.04	0.02	0.07	0.00013195299	0.001
Stringency index (14 lags)	-0.07	-0.10	-0.05	0.00000005759	0.001
**Full model, with workplaces**					
Effective vaccine coverage	-0.30	-0.46	-0.15	0.00022243816	0.001
Mobility: workplaces (14 lags)	0.02	0.00	0.03	0.01410526340	0.001
Stringency index (14 lags)	-0.09	-0.11	-0.07	0.00000000001	0.001

Note: the adjusted *P*-values are the False Discovery Rate (FDR) *Q*-values computed using Anderson's code, available at: https://are.berkeley.edu/~mlanderson/downloads/fdr_sharpened_qvalues.do.zip.

Appendix S9 Mobility model: adding country socioeconomic development status (SDS) as moderators

To explore socioeconomic factors that may change the relationship between vaccine coverage and human mobility, we focused on eight nonmonetary measures of socioeconomic development status (SDS) in four dimensions: human capital, social capital, employment, and digitalization. The measurement, definition and data sources are summarised in Table S9.1. The hypothesis is that, compared with countries with lower SDS, those with higher SDS may be more likely to maintain social distancing after COVID-19 vaccination.

The first dimension, education, is closely related to a country's human capital. Education is also a key dimension of human development, and could affect human behaviour [1]. For this dimension, we used the secondary rather than primary school enrolment, because it had a larger variation across countries than primary school enrolment did. The second dimension, social capital, is defined as "networks together with shared norms, values and understandings that facilitate cooperation within or among groups" [2]. The World Bank similarly describes social capital as "the institutions, relationships, and norms that shape the quality and quantity of a society's social interactions" [3]. Social capital is likely to contribute to collective preventive behaviour, where trust is a key element. Especially, in the absence of lockdown policies, social cohesion could foster compliance of social distancing. We focused on trust science and trust people in neighbourhood, since these factors may influence decisions on social distancing.

The third dimension considered different occupation and employment status. Level of required physical attendance varied across occupation, which may lead to varying degrees of mobility. Self-employed work status has been widely used to describe workers' behaviour and socioeconomic groups [4]. A sizeable self-employed workers could be an indication of a large agriculture sector and low growth of formal economy [4]. Finally, we included the dimension of digitalization because people with better digital capability may be more able to maintain physical and social distancing in daily life.

We calculated the mean value of individual SDS variables for individual income groups. The results are presented in Table S9.2. Jame's test [5] rejected the null hypothesis that the mean is equal across income group. Compared with lower- and upper-middle income countries, high-income countries appeared to have a higher enrolment ratio, higher trust, a larger share of employment in the industry and service sector, a much smaller share of workers in the agriculture sector and self-employed status, and a substantially larger share of individuals using the internet. Some countries did not report related data. Therefore, the sample size decreased from 107 to 79 and 91 countries for school enrolment and trust, respectively.

We added eight SDS variables to the mobility model one-by-one and re-ran the regression. To investigate whether they were moderators, an interaction term between the SDS and the vaccine coverage, was included. The results were presented in Table S9.3. The random-effect model showed that the interaction was statistically significant (P<0.001) for all SDS in all four location categories, suggesting that the magnitude of vaccination-induced mobility (VM) varied with SDS.

To understand the association of vaccine coverage with mobility at different values of SDS, we estimated the average marginal effect of vaccine coverage at the 10th, 30th, 50th, 70th and 90th percentile of distribution of each SDS. The results are displayed in Table S9.4. Consider, for example, the first column of school enrolment. The interpretation of dy/dx was that, when the enrolment ratio was 45.6%, 54.5%, 61.7%, 73.6% and 78.9%, a 10 percentage points increase in vaccine coverage was associated with a 5.58, 4.18, 3.81, 3.58 and 2.52 percentage points (P<0.001) increase in the number of visitors to retail stores, respectively. The most substantial difference in the marginal effect was observed for digitalization. The VM in grocery stores was 11.36 and 2.08 percentage points, respectively, when the share of internet users shifted from the 10th to the 90th percentile of the distribution. This result implied the critical role of digital capability to hold back human mobility after vaccination. In sum, the key findings are that, the magnitude of VM were smaller for countries with

higher school enrolment ratios, trust in science, or trust in people; larger shares of employment in industry or service sectors or internet users, and smaller shares of agriculture employment or self-employed. Overall, the marginal effect analysis supported our conjecture that countries with higher SDS had lower VM.

References:

1. UNDP. 2022. Human Development Report 2021-22. New York: United Nations Development Programme.

2. OECD. The well-being of nations: the role of human and social capital, OECD, Paris. Scrivens, K. and C. Smith (2013), Four interpretations of social capital: an agenda for measurement, OECD statistics working papers. Available from: https://doi.org/10.1787/5jzbcx010wmt-en [accessed 22 Jan. 2023].

3. The World Bank. What is social capital? Available from: https://web.worldbank.org/archive/website01360/WEB/0__CO-10.HTM [accessed 22 Jan. 2023].

4. International Labour Organization. 2015. KILM 3. Status in employment. Available from: https://www.ilo.org/wcmsp5/groups/public/---dgreports/---stat/documents/publication/wcms_422401.pdf [accessed 22 Jan. 2023].

5. James GS. Tests of linear hypotheses in univariate and multivariate analysis when the ratios of the population variances are unknown. Biometrika. 1954;41:19-43.

Table S9.1 Summary of variables for country socioeconomic development status

	Measurement	Definition
**I. Human capital**[a]		
School enrolment, secondary	% gross	The ratio of total enrolment (regardless of age), to the population of the age group that officially corresponds to the level of education. The ratio includes overage and underage students.
**II. Social capital**[b]		
Trust science	From 0 (low) to 100 (high)	Survey questions: "How much do you trust each of the following: other people in your neighbourhood; your national government; scientists; journalists; doctors and nurses; people who work at non-governmental or non-profit organizations; healers? Do you trust them a lot, some, not much, or not at all?"
Trust people in neighbourhood	From 0 (low) to 100 (high)	
**III. Employment**[c]		
Employment in industry	From 0 to 100 (% of total employment)	The industry sector consists of: mining and quarrying; manufacturing; construction; public utilities (electricity, gas, and water).
Employment in service	From 0 to 100 (% of total employment)	The services sector consists of:<br>1. Wholesale, retail trade, restaurants and hotels<br>2. Transport, storage, and communications<br>3. Financing, insurance, real estate, and business services<br>4. Community, social, and personal services
Employment in agriculture	From 0 to 100 (% of total employment)	The agriculture sector consists of activities in agriculture, hunting, forestry and fishing,
Self-employed	From 0 to 100 (% of total employment)	Self-employed workers are those workers who, working on their own account or with one or a few partners or in cooperative, hold the type of jobs defined as a "self-employment jobs.", such as contributing family workers.
**IV. Digitalization**		
Individuals using the internet[d]	From 0 to 100 (% of population)	Internet users are individuals who have used the Internet (from any location) in the last 3 months, via a computer, mobile phone, personal digital assistant, games machine, digital TV etc.

a. UNESCO Institute for Statistics (UIS). UIS Developer Portal. Bulk Data Download Service. Data source: https://apiportal.uis.unesco.org/bdds [accessed 22 Jan. 2023].

b. Wellcome Global Monitor (2020). Data source: https://wellcome.org/reports/wellcome-global-monitor-covid-19/2020 [accessed 22 Jan. 2023].

c. International Labour Organization. “ILO modelled estimates database” ILOSTAT. Data source: https://ilostat.ilo.org/data/ [accessed 22 Jan. 2023].

d. International Telecommunication Union World Telecommunication/ICT Indicators Database. Data source: https://data.worldbank.org/indicator/IT.NET.USER.ZS [accessed 22 Jan. 2023].

Table S9.2 Mean value for country socioeconomic development status (SDS), by income group

SDS / Income group	School enrolment, secondary (% gross)	Trust Science (0-100)	Trust people in neighbourhood (0-100)	Employment in industry (% of total employment)	Employment in services (% of total employment)	Employment in agriculture (% of total employment)	Self-employed (% of total employment)	Individuals using the internet (% of population)
**High income**	112.7	87.3	79.9	23.6	72.1	4.3	15.1	85.4
**Upper-middle income**	98.5	70.7	63.3	21.5	61.5	17.0	31.8	65.9
**Lower-middle income**	74.6	73.7	68.5	19.1	44.4	36.5	56.1	38.1
Test for equality of three group means	$\chi^2(2)$ = 40095.4; Prob >χ^2 =0.0000	$\chi^2(2)$ = 45629.1; Prob > χ^2 =0.0000	$\chi^2(2)$ = 29974.5; Prob > χ^2 =0.0000	$\chi^2(2)$ = 5273.4; Prob > χ^2 =0.0000	$\chi^2(2)$ = 95987.8; Prob > χ^2 =0.0000	$\chi^2(2)$ = 128985.6; Prob > χ^2 =0.0000	$\chi^2(2)$ = 115712.0; Prob > χ^2 =0.0000	$\chi^2(2)$ = 177572.9; Prob > χ^2 =0.0000
Year	2018	2020	2020	2018	2018	2018	2018	2018
No. of countries	79	91	91	106	106	106	106	102

Note: Jame’s test which allows heterogeneous covariance matrices across income groups was conducted.

Table S9.3 Results from mobility model: adding SDS and interaction with vaccine coverage

Y: daily mobility change (%)	Retail & recreation<br>Coef.	Grocery & pharmacy<br>Coef.	Transit stations<br>Coef.	Workplaces<br>Coef.
No. of people vaccinated (per ten people)	9.74***	14.07***	10.21***	5.13***
School enrolment ratio (secondary, % gross)	0.01	0.07	-0.05	-0.03
No. of people vaccinated × School enrolment ratio	-0.06***	-0.09***	-0.07***	-0.04***
N	*56,113*	*56,116*	*56,129*	*56,126*
No. of people vaccinated (per ten people)	13.41***	20.20***	13.76***	7.83***
Trust science	-0.10	0.06	-0.07	0.13
No. of people vaccinated × Trust science	-0.12***	-0.19***	-0.13***	-0.08***
N	*64,470*	*64,484*	*64,464*	*64,404*
No. of people vaccinated (per ten people)	10.51***	15.23***	10.91***	6.56***
Trust people in neighbourhood	-0.01	0.12	-0.11	0.12
No. of people vaccinated × Trust people in neighbourhood	-0.09***	-0.15***	-0.11***	-0.07***
N	*64,470*	*64,484*	*64,464*	*64,404*
No. of people vaccinated (per ten people)	5.43***	5.98***	3.79***	2.46***
Employment in industry	-0.13	0.42	-0.01	0.10
No. of people vaccinated × Employment in industry	-0.08**	-0.07*	-0.04	-0.05**
N	*75,005*	*74,995*	*74,940*	*74,949*
No. of people vaccinated (per ten people)	9.25***	13.55***	8.28***	4.15***
Employment in service	0.14	0.22	-0.05	-0.13
No. of people vaccinated × Employment in service	-0.08***	-0.14***	-0.08**	-0.04*
N	*75,005*	*74,995*	*74,940*	*74,949*
No. of people vaccinated (per ten people)	2.37***	2.42***	1.62***	0.75***
Employment in agriculture	-0.06	-0.27	0.07	0.10
No. of people vaccinated × Employment in agriculture	0.12***	0.18***	0.11**	0.06**
N	*75,005*	*74,995*	*74,940*	*74,949*
No. of people vaccinated (per ten people)	1.40***	1.08*	0.77	0.19
Self-employment	-0.08	-0.13	0.03	0.03
No. of people vaccinated × Self-employment	0.09***	0.14***	0.08***	0.05***
N	*75,005*	*74,995*	*74,940*	*74,949*
No. of people vaccinated (per ten people)	11.51***	16.30***	10.17***	5.58***
Individuals using the internet	-0.01	0.03	-0.08	-0.11
No. of people vaccinated × Individuals using the internet	-0.10***	-0.15***	-0.10***	-0.05***
N	*72,195*	*72,185*	*72,130*	*72,139*

Note: Random-effects models were applied. The included common covariates not shown in the table were stringency index, risk information (as measured by the lagged number of new cases), population density, the share of people aged 65 or over, GDP per capita, Gini index, % female population, and indicators for continent and season. Standard errors were clustered at the country level.

Legend: * P <0.05; ** P < 0.01; *** P < 0.001

Table S9.4 Average marginal effects of vaccine coverage on mobility, at different values of SDS

Y: daily mobility change (%)	**Retail and recreation**			**Grocery and pharmacy**			**Transit stations**			**Workplaces**		
	dy/dx	SE	*P*>z	dy/dx	SE	*P*>z	dy/dx	SE	*P*>z	dy/dx	SE	*P*>z
No. of people vaccinated (per ten people) at:												
School enrolment ratio =45.6 (p10)	5.58	0.62	<0.001	7.38	0.71	<0.001	5.17	0.58	<0.001	2.58	0.43	<0.001
School enrolment ratio =54.5 (p30)	4.18	0.32	<0.001	5.13	0.38	<0.001	3.48	0.34	<0.001	1.72	0.22	<0.001
School enrolment ratio =61.7 (p50)	3.81	0.27	<0.001	4.53	0.32	<0.001	3.02	0.30	<0.001	1.49	0.18	<0.001
School enrolment ratio =73.6 (p70)	3.58	0.25	<0.001	4.17	0.30	<0.001	2.75	0.28	<0.001	1.35	0.16	<0.001
School enrolment ratio =78.9 (p90)	2.52	0.35	<0.001	2.46	0.38	<0.001	1.47	0.32	<0.001	0.70	0.19	0.001
	dy/dx	SE	*P*>z	dy/dx	SE	*P*>z	Coef.	SE	*P*>z	dy/dx	SE	*P*>z
No. of people vaccinated (per ten people) at:												
Trust Science = 64.1 (p10)	5.85	0.71	<0.001	7.95	0.99	<0.001	5.29	0.73	<0.001	2.83	0.48	<0.001
Trust Science = 75.9 (p30)	4.46	0.37	<0.001	5.69	0.52	<0.001	3.73	0.40	<0.001	1.91	0.25	<0.001
Trust Science = 80.8 (p50)	3.88	0.26	<0.001	4.75	0.37	<0.001	3.08	0.31	<0.001	1.52	0.17	<0.001
Trust Science = 86.2 (p70)	3.24	0.22	<0.001	3.72	0.31	<0.001	2.37	0.30	<0.001	1.02	0.15	<0.001
Trust Science = 93.4 (p90)	2.39	0.34	<0.001	2.34	0.46	<0.001	1.42	0.43	<0.001	0.54	0.22	0.002
	dy/dx	SE	*P*>z	dy/dx	SE	*P*>z	dy/dx	SE	*P*>z	dy/dx	SE	*P*>z
No. of people vaccinated (per ten people) at:												
Trust People in neighbourhood = 55.2 (p10)	5.41	0.49	<0.001	7.17	0.62	<0.001	4.90	0.57	<0.001	2.70	0.28	<0.001
Trust People in neighbourhood = 66.3 (p30)	4.39	0.30	<0.001	5.55	0.40	<0.001	3.69	0.36	<0.001	1.93	0.18	<0.001
Trust People in neighbourhood = 74.2 (p50)	3.66	0.22	<0.001	4.39	0.31	<0.001	2.83	0.27	<0.001	1.38	0.14	<0.001
Trust People in neighbourhood = 81.1 (p70)	3.02	0.24	<0.001	3.38	0.32	<0.001	2.08	0.28	<0.001	0.90	0.15	<0.001
Trust People in neighbourhood = 87.9 (p90)	2.39	0.33	<0.001	2.39	0.41	<0.001	1.34	0.36	<0.001	0.42	0.19	0.030

Table S9.4 Average marginal effects of vaccine coverage on mobility, broken down by country socioeconomic development status (continued)

Y: daily mobility change (%)	Retail and recreation			Grocery and pharmacy			Transit stations			Workplaces		
	dy/dx	SE	*P*>z	dy/dx	SE	*P*>z	dy/dx	SE	*P*>z	dy/dx	SE	*P*>z
No. of people vaccinated (per ten people) at:												
Employment in industry = 14.23 (p10)	4.31	0.43	<0.001	4.94	0.55	<0.001	3.15	0.52	<0.001	1.79	0.26	<0.001
Employment in industry = 18.81 (p30)	3.96	0.33	<0.001	4.61	0.42	<0.001	2.94	0.38	<0.001	1.58	0.20	<0.001
Employment in industry = 20.82 (p50)	3.80	0.29	<0.001	4.47	0.37	<0.001	2.85	0.33	<0.001	1.48	0.18	<0.001
Employment in industry = 24.85 (p70)	3.48	0.22	<0.001	4.17	0.30	<0.001	2.67	0.25	<0.001	1.29	0.15	<0.001
Employment in industry = 30.11 (p90)	3.07	0.21	<0.001	3.79	0.30	<0.001	2.44	0.24	<0.001	1.05	0.17	<0.001
	dy/dx	SE	*P*>z	dy/dx	SE	*P*>z	dy/dx	SE	*P*>z	dy/dx	SE	*P*>z
No. of people vaccinated (per ten people) at:												
Employment in service = 42.11 (p10)	5.71	0.75	<0.001	7.73	1.21	<0.001	4.80	0.85	<0.001	2.42	0.52	<0.001
Employment in service = 56.14 (p30)	4.53	0.43	<0.001	5.79	0.67	<0.001	3.64	0.48	<0.001	1.84	0.30	<0.001
Employment in service = 63.39 (p50)	3.93	0.29	<0.001	4.79	0.42	<0.001	3.04	0.33	<0.001	1.54	0.20	<0.001
Employment in service = 71.08 (p70)	3.28	0.22	<0.001	3.73	0.26	<0.001	2.41	0.27	<0.001	1.23	0.15	<0.001
Employment in service = 78.90 (p90)	2.62	0.30	<0.001	2.64	0.39	<0.001	1.76	0.38	<0.001	0.90	0.21	<0.001
	dy/dx	SE	*P*>z	dy/dx	SE	*P*>z	dy/dx	SE	*P*>z	dy/dx	SE	*P*>z
No. of people vaccinated (per ten people) at:												
Employment in agriculture = 1.37 (p10)	2.54	0.26	<0.001	2.67	0.33	<0.001	1.77	0.32	<0.001	0.84	0.18	<0.001
Employment in agriculture = 3.76 (p30)	2.83	0.23	<0.001	3.11	0.27	<0.001	2.03	0.28	<0.001	0.99	0.16	<0.001
Employment in agriculture = 9.62 (p50)	3.55	0.22	<0.001	4.18	0.29	<0.001	2.68	0.27	<0.001	1.36	0.16	<0.001
Employment in agriculture = 21.66 (p70)	5.02	0.46	<0.001	6.38	0.73	<0.001	4.00	0.56	<0.001	2.12	0.35	<0.001
Employment in agriculture = 38.7 (p90)	7.09	0.92	<0.001	9.49	1.47	<0.001	5.87	1.10	<0.001	3.19	0.70	<0.001

Table S9.4 Average marginal effects of vaccine coverage on mobility, broken down by country socioeconomic development status (continued)

Y: daily mobility change (%)	**Retail and recreation**			**Grocery and pharmacy**			**Transit stations**			**Workplaces**		
	dy/dx	SE	*P*>z	dy/dx	SE	*P*>z	dy/dx	SE	*P*>z	dy/dx	SE	*P*>z
No. of people vaccinated (per ten people) at:												
Self-employment = 8.45 (p10)	2.19	0.28	<0.001	2.23	0.38	<0.001	1.47	0.34	<0.001	0.62	0.19	0.001
Self-employment = 15.03 (p30)	2.81	0.23	<0.001	3.12	0.29	<0.001	2.02	0.27	<0.001	0.96	0.15	<0.001
Self-employment = 25.05 (p50)	3.74	0.22	<0.001	4.47	0.31	<0.001	2.85	0.28	<0.001	1.46	0.16	<0.001
Self-employment = 39.34 (p70)	5.08	0.37	<0.001	6.40	0.57	<0.001	4.08	0.48	<0.001	2.19	0.29	<0.001
Self-employment = 64.37 (p90)	7.42	0.74	<0.001	9.78	1.15	<0.001	6.13	0.95	<0.001	3.45	0.57	<0.001
	dy/dx	SE	*P*>z	dy/dx	SE	*P*>z	dy/dx	SE	*P*>z	dy/dx	SE	*P*>z
No. of people vaccinated (per ten people) at:												
Individuals using the internet = 31.9 (p10)	8.26	0.82	<0.001	11.36	1.25	<0.001	7.13	1.02	<0.001	3.87	0.63	<0.001
Individuals using the internet = 59.6 (p30)	5.44	0.40	<0.001	7.07	0.60	<0.001	4.50	0.50	<0.001	2.38	0.31	<0.001
Individuals using the internet = 73.48 (p50)	4.03	0.24	<0.001	4.92	0.32	<0.001	3.17	0.30	<0.001	1.63	0.18	<0.001
Individuals using the internet = 83.00 (p70)	3.06	0.21	<0.001	3.44	0.25	<0.001	2.27	0.26	<0.001	1.12	0.15	<0.001
Individuals using the internet = 91.80 (p90)	2.16	0.27	<0.001	2.08	0.34	<0.001	1.43	0.33	<0.001	0.65	0.19	0.001

Note: Random-effects models were applied. The marginal effect was noted by dy/dx, and estimated from coefficients presented in Table S8.2. Standard errors were clustered at the country level and estimated by using the Delta-method.
